# Underlining the Importance of Peripheral Protic Functional Groups to Enhance the Proton Exchange of Gd-Based MRI Contrast Agents

**DOI:** 10.1021/acs.inorgchem.1c01927

**Published:** 2021-08-13

**Authors:** Mariangela Boccalon, Loredana Leone, Giuseppe Marino, Nicola Demitri, Zsolt Baranyai, Lorenzo Tei

**Affiliations:** † Bracco Research Centre, Bracco Imaging S.p.A., Via Ribes 5, 10010 Colleretto Giacosa, Italy; ‡ Dipartimento di Scienze e Innovazione Tecnologica, Università del Piemonte Orientale “A. Avogadro”, Viale T. Michel 11, 15121 Alessandria, Italy; § Elettra−Sincrotrone Trieste, S.S. 14 Km 163.5 in Area Science Park, Basovizza, 34149 Trieste, Italy

## Abstract

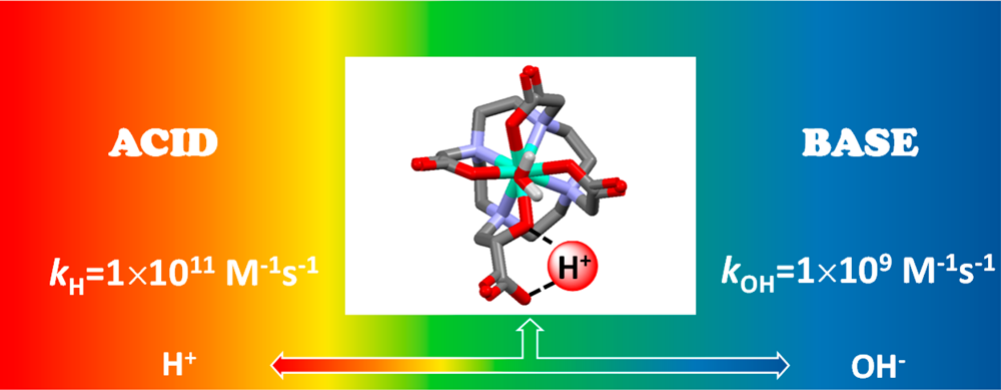

In this study, we report the synthesis and the equilibrium, kinetic, relaxation, and structural properties of two new Gd^III^ complexes based on modified 10-(2-hydroxypropyl)-1,4,7,10-tetraazacyclododecane-1,4,7-triacetic acid (HPDO3A) designed to modulate the relaxivity at acidic and basic pH due to intra- and intermolecular proton exchange. The presence of a carboxylic or ester moieties in place of the methyl group of HPDO3A allowed differentiation of a protic and nonprotic functional group, highlighting the importance of the formation of an intramolecular hydrogen bond between the coordinated hydroxyl and the carboxylate groups for proton exchange (*k*_H_ = 1.5 × 10^11^ M^–1^ s^–1^, *k*_OH_ = 1.7 × 10^9^ M^–1^ s^–1^). The determination of the thermodynamic stability and kinetic inertness of the Gd^III^ complexes confirmed that the modification of peripheral groups does not significantly affect the coordination environment and thus the stability (log *K*_GdL_ = 19.26, *t*_1/2_ = 2.14 × 10^7^ hours, pH = 7.4, 0.15 M NaCl, 25 °C). The relaxivity (*r*_1_) was measured as a function of pH to investigate the proton exchange kinetics, and as a function of the magnetic field strength to extrapolate the relaxometric parameters (*r*_1_^GdL1^ = 4.7 mM^–1^ s^–1^ and *r*_1_^GdL2^ = 5.1 mM^–1^ s^–1^ at 20 MHz, 25 °C, and pH 7.4). Finally, the X-ray crystal structure of the complex crystallized at basic pH showed the formation of a tetranuclear dimer with alkoxide and hydroxide groups bridging the Gd^III^ ions.

## Abstract 2

The peripheral carboxylic moiety of a 2-hydroxypropanoic pendant arm of a GdHPDO3A-like complex forms an intramolecular hydrogen bond with the −OH group that allows both acid- and base-catalyzed proton exchange and thus a relaxivity enhancement. Conversely, the nonprotic ester group in the same position permits only the base-catalyzed mechanism.

## Introduction

Paramagnetic gadolinium chelates were introduced several decades ago as medical magnetic resonance imaging (MRI) contrast agents (GBCAs) to enhance the differences between normal and diseased tissues by markedly accelerating the relaxation rates of the hydrogen atoms of the body fluids.^1,2^ During the past two decades, the great relevance of MRI in modern diagnostic medicine has driven the search for GBCA optimization by modulation of the main parameters governing paramagnetic relaxation, i.e., the number (*q*) and residence lifetime (τ_M_ = 1/*k*_ex_) of the metal-coordinated water molecule(s) and the rotational motion of the paramagnetic system, described by the correlation time τ_R_.^3,4^ Another process effective in enhancing the nuclear relaxation rate of solvent water protons (relaxivity, *r*_1_) is the exchange with the bulk water of the mobile protons present at a relatively short distance from the Gd^III^ center.^5−8^

**Scheme 1:**
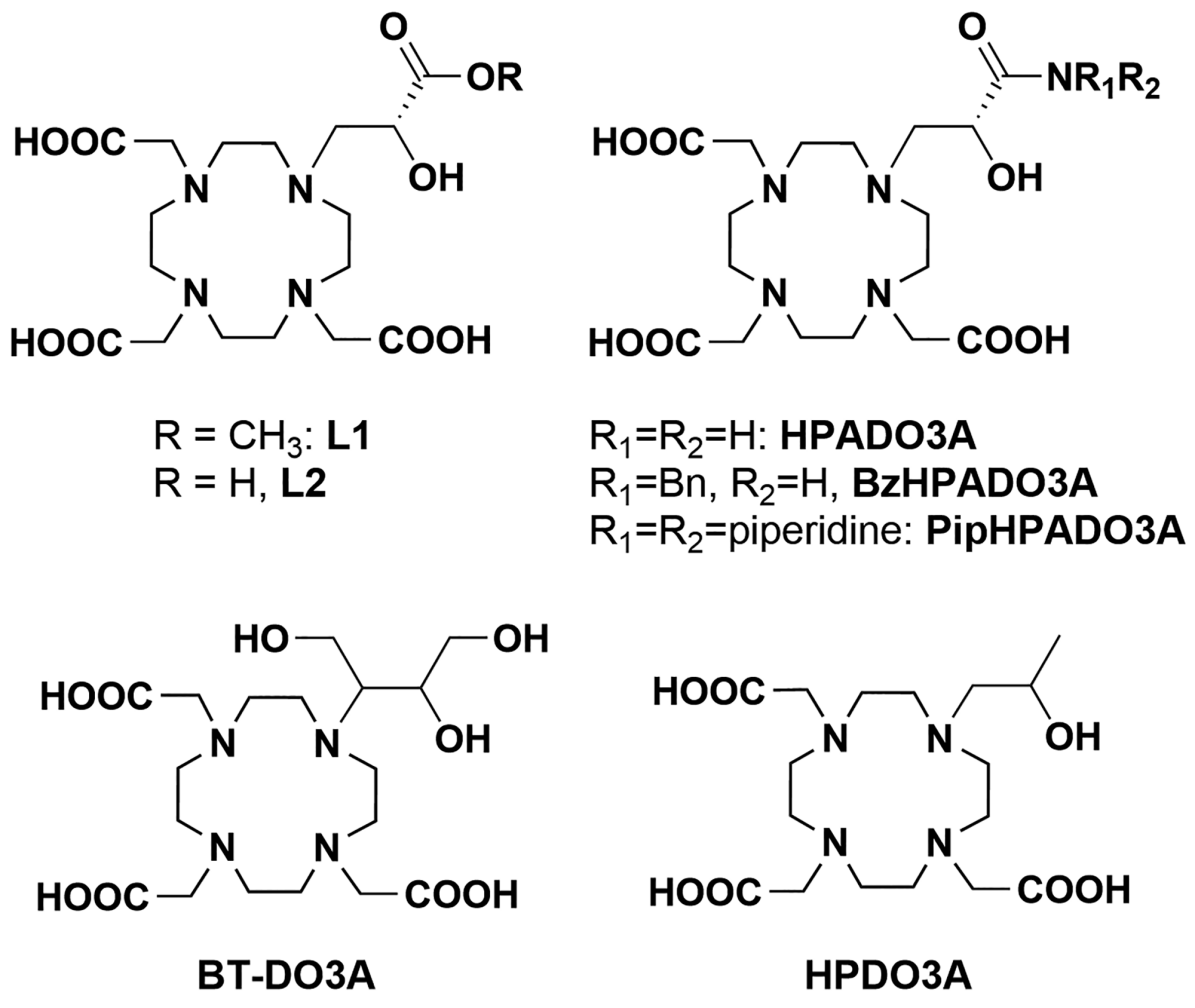
Chelating Ligand Discussed in the Present Work

This proton exchange has been highlighted in the case of GdHPDO3A (HPDO3A = 10-(2-hydroxypropyl)-1,4,7,10-tetraazacyclododecane-1,4,7-triacetic acid, Scheme 1) where a hydroxyl group is coordinated to the metal center. Thus, the hydroxyl proton is in fast exchange with bulk water at high pH values (pH > 10) providing a substantial base-catalyzed proton exchange contribution to *r*_1_ (*Δr*_1_ = 1.2 mM^–1^ s^–1^).^5^ The fast proton exchange of the OH group has also been shown to slightly increase *r*_1_ at neutral pH in the presence of the basic component of buffers (e.g., phosphate, carbonate, and HEPES).^5^ In addition, the modulation of the chemical groups located in place of the methyl group of HPDO3A has led to *r*_1_ enhancement due to several peculiar properties.^6−8^ For example, hydroxyl-, amino-, or carboxy-benzyl groups have been shown to favor intramolecular H-bonding with the coordinated hydroxyl moiety, affecting both the p*K* values of the involved functionalities and the rate of the proton exchange process.^6^ An even more remarkable effect has been featured by amide functionalities (GdHPADO3As, Scheme 1) that provide labile protons capable of establishing an acid-catalyzed proton exchange process with the metal-coordinated OH group and second sphere water molecules causing a remarkable relaxivity increase (*Δr*_1_ = 5.5 mM^–1^ s^–1^ from pH 7.4 to 5 for GdHPADO3A).^7^ The importance of introducing functional groups at the periphery of Gd^III^ complexes in the correct position to form hydrogen bonds with the coordinated and/or second sphere water molecules has also been recently demonstrated providing either a relaxivity increase or modulation of the water exchange rate.^9−11^

In this work, we broadened the study on HPDO3A derivatives containing a carbonyl group in place of the methyl in the hydroxypropyl arm of HPDO3A by inserting a carboxylic or an ester group instead of the amide group present in the HPADO3A derivatives. In order to broaden the pH range of the proton exchange without the use of external catalysts, we aimed to investigate the effect of the carboxylic acid proton in the proximity of the coordinated hydroxyl group on the proton exchange relaxation enhancement. Moreover, the effect of the presence of a free carboxylate group in forming an H-bonded network of second sphere water molecules or possible dimerization, as already shown recently for a Gd^III^-DO3A-sulfonamide derivative bearing a peripheral carboxylate,^12^ will be examined. Thus, two new GdHPDO3A-like complexes containing an ester or a carboxylic acid (GdL1 and GdL2, Scheme 1) were synthesized and investigated by ^1^H NMR relaxometry as well as solution thermodynamic and kinetic studies. Finally, the formation of a dimeric tetranuclear complex {[(Gd(H_2_O)_2_)_2_[Gd(L2)H_–1_(HO^–^)]_2_}_2_ at basic pH with a slight excess of Gd(OH)_3_ was elucidated by single crystal X-ray diffraction analysis.

### Synthesis

**Scheme 2:**
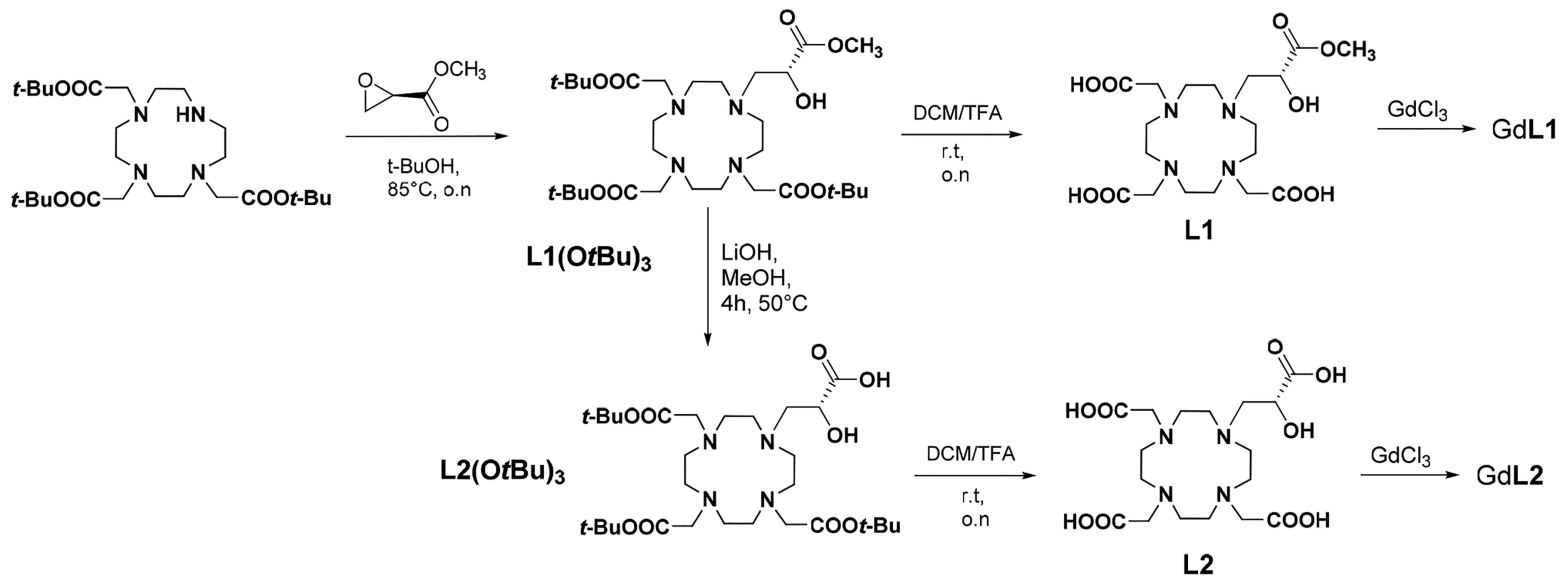
Synthesis of Ligands L1 and L2 and Their Gd(III) Complexes

The ligand L1 was obtained from the ring opening of methyl (2*R*)-glycidate with the secondary amine of DO3A(*t*BuO)_3_ to obtain L1(O*t*Bu)_3_,^7^ followed by deprotection of the *t*-butyl esters with trifluoroacetic acid (TFA) and dichloromethane (DCM) (Scheme 2). On the other hand, the ligand L2 was prepared by hydrolysis of the methyl ester in L1(O*t*Bu)_3_ followed by deprotection of the *t*-butyl esters by TFA/DCM (1:1). The Gd^III^ complexes were then obtained in aqueous solution at pH = 7.0 by the reaction of the ligand with a stoichiometric amount of GdCl_3_.

### Equilibrium and Kinetic Characterizations

The metal complexes of biomedical interest must show a high *in vivo* stability, which includes high thermodynamic stability and kinetic inertness to allow targeting applications and to avoid possible toxic effects derived by metal ion and ligand release via transmetalation or transchelation reactions.^2,3,13^ The protonation constants of L2 as well as the stability and the protonation constants of GdL2 and those of the most important endogenous divalent metal complexes (Ca^II^-, Zn^II^-, and Cu^II^) were determined by pH potentiometry (Table 1 and Table S1). In the case of GdL1, we were interested in determining the dissociation constant of the hydroxyl group that was measured also by pH potentiometry (Table 1). Experimental details and the definitions and equations used for the evaluation of the equilibrium and kinetic data are summarized in the SI.

**Table 1:** Protonation Constants of L2: Stability and Protonation Constants of Ca^II^, Zn^II^, Cu^II^, and Gd^III^ Complexes Formed with L2 Compared with Literature Data on HPADO3A, BT-DO3A, and DOTA Ligands and the Rate Constants (*k*_i_) and Half-Lives (*t*_1/2_ = ln 2/*k*_d_) Characterizing the Dissociation Reactions of GdL2, GdHPADO3A, GdDOTA, GdHPDO3A, and GdBT-DO3A Complexes (298 K)

	L2	HPADO3A^a^	HPDO3A^b^^,^^c^	DOTA^d^	BT-DO3A^e^
*I*	0.15 M NaCl		0.1 M Me_4_NCl	0.1 M NaCl	0.1 M NaCl
log *K*_1_^H^	8.95 (3)	8.96	11.96	9.37	9.46
log *K*_2_^H^	8.95 (2)	9.07	9.43	9.14	9.36
log *K*_3_^H^	4.22 (3)	4.22	4.30	4.63	4.17
log *K*_4_^H^	3.74 (3)	2.64	3.26	3.91	3.02
log *K*_5_^H^	2.47 (4)	1.25			
log *K*_5_^H^	1.75 (4)				
CaL	11.63 (1)	12.13	14.83	16.37^f^	12.1
ZnL	17.81 (6)	17.18	19.37	18.7^f^	17.0
CuL^g^	21.87 (6)	21.53	22.84	22.72^f^	19.1
GdL	19.26 (3)	18.41	23.8	24.7	18.7
Gd(HL)	3.36 (3)				
Gd(L)H_–1_	9.58 (3)	6.73	11.36^e^		9.48
pGd	16.87^h^	16.88^h^	18.16^h^	22.09^h^	15.63^h^

	GdL2	GdHPADO3A	GdHPDO3A^i^	GdDOTA^i^	GdBT-DO3A
*k*_1_^j^ (M^–1^ s^–1^)	(2.3 ± 0.1) × 10^–4^	1.6 × 10^–4^	2.9 × 10^–4^	1.8 × 10^–6^	2.8 × 10^–5^
*K*^H^_GdL_ (M^–1^)	0.5 ± 0.1 (GdHL)			14	
*k*_d_ (s^–1^) at pH = 7.4	9.01 × 10^–12^	6.41 × 10^–12^	1.15 × 10^–11^	7.28 × 10^–14^	1.35 × 10^–12^
*t*_1/2_ (h) at pH = 7.4	2.14 × 10^7^	3.00 × 10^7^	1.67 × 10^7^	2.64 × 10^9^	1.42 × 10^8^

a Ref (7).

b Ref (19).

c Ref (20).

d Ref (22).

e Ref (15).

f Ref (21); 0.1 M KCl, 25 °C.

g Spectrophotometry, *I* = [Na^+^]+[H^+^] = 0.15 M, [H^+^] ≤ 0.15 M;

h pGd = −log[Gd]_free_, [Gd^3+^] = 1 μM, [L] = 10 μM, pH = 7.4 (ref (23)). GdL1: log *K*_Gd(L)H-1_ = 9.36 (6), 0.15 M NaCl, 298 K.

i Ref (18).

j *k*_1_ = *k*_GdH2L_ × *K*^H^_Gd(HL)_.

As shown in Table 1, the stability constants of Gd^III^, Ca^II^, Zn^II^, and Cu^II^ complexes formed with L2, HPADO3A, and BT-DO3A ligands (Scheme 1) are very similar and about 1–5 orders of magnitude smaller than those of the corresponding DOTA and HPDO3A complexes. It should be noted that the equilibrium data reported in Table 1 were determined using solutions of different ionic strengths; therefore, the lower stability of the Gd^III^ complexes with L2, HPADO3A, and BT-DO3A compared to GdHPDO3A can be explained by the formation of Na^+^ complexes as already highlighted in the case of DOTA derivatives (log *K*_Na(DOTA)_ = 4.38; log *K*_Na(BT-DO3A)_ = 2.32).^14,15^ On the other hand, Table 1 also shows that the stability constant of GdDOTA is about 5 orders of magnitude higher than those of GdL2, GdHPADO3A, and GdBT-DO3A, which can be the consequence of the stronger electrostatic metal–ligand interaction of the Gd^III^ ion with the four negatively charged carboxylate groups of the DOTA ligand as compared to those with the three carboxylate groups of L2, HPADO3A, and BT-DO3A. The protonation constants of the alkoxide O^–^ in GdL1 and GdL2 complexes are lower than in the case of GdHPDO3A (log *K*_Gd(L)H-1_ = 11.34, Table 1), due to the electron withdrawing effect of the carbonyl group, and somewhat higher than that of GdHPADO3A, due to the lower stabilization of the alkoxide anion by the carboxylate (GdL2) or the ester (GdL1) groups with respect to the amide function. On the other hand, the log *K*_Gd(L)H-1_ value of GdL2 is slightly higher than that of GdL1 due to the H-bond formation between the deprotonated carboxylate and the alcoholic −OH group. The protonation constants characterizing the acid–base properties of the carboxylate group on the 2-hydroxypropanoic pendant of the free L2 ligand and GdL2 complex (Table 1 and Table S1) are essentially identical, which confirms that the carboxylate group of the 2-hydroxypropanoic arm does not coordinate the Gd^III^ ion. The similar stability of GdL2 and GdHPADO3A complexes is also confirmed by the conditional stability constants (pGd), which is about one unit higher than that of GdBT-DO3A.

In order to investigate the kinetic inertness of the Gd^III^ complex, the dissociation reactions of GdL2 were followed by ^1^H NMR relaxometry (21 MHz and 298 K) in the presence of large acid excess ([HCl] = 0.01–1.0 M) to guarantee the pseudo-first-order kinetic conditions. Pseudo-first-order rate constants (*k*_d_) increase with increasing concentration of H^+^ (Figure S4) due to the proton-assisted dissociation of GdL2 (*k*_1_) via the formation of the protonated *Gd(H_2_L2) intermediate (*K*_GdH2L_). The second proton is presumably attached to one coordinated carboxylate group of GdL2. Based on the kinetic parameters reported in Table 1, the rates of the acid-catalyzed dissociation (*k*_1_) of GdL2 and GdHPADO3A are very similar and about 10 and 100 times higher than those of GdBT-DO3A and GdDOTA, respectively. Since the dissociation of LnDOTA-like complexes generally occurs via the proton-assisted pathway without the essential metal ion-assisted (e.g., Ca^II^, Zn^II^, Cu^II^, and Fe^III^) reactions,^15−18^ the dissociation rate (*k*_d_) and half-life values (*t*_1/2_ = ln 2/*k*_d_) of Gd^III^ complexes were calculated by considering only the contribution of the acid-assisted dissociation close to physiological conditions (pH = 7.4, 298 K). Thus, the *t*_1/2_ value for GdL2 is comparable to that of GdHPDO3A and slightly lower than that of GdHPADO3A, which may be due to the presence of the protonable, noncoordinated carboxylate group, which can promote the release of the Gd^III^ ion via the proton transfer to the macrocyclic N atom.

### Relaxometric Characterization

The longitudinal relaxivity values (*r*_1_) for GdL1 and GdL2, at 20 MHz (0.47 T), 298 K, and pH 7.4, are 4.7 and 5.1 mM^–1^ s^–1^, respectively. These are typical values of the clinical MRI contrast agents, i.e., low-molecular-weight *q* = 1 Gd^III^ chelates that tumble rapidly in solution.^1,2^ In particular, these values compare well with the *r*_1_ reported for GdHPDO3A^24^ and other HPDO3A-like complexes such as GdHPDO3MA^25^ or the series of Gd-hydroxypropylamide-DO3A (HPADO3A, Scheme 1)^7^ complexes measured at the same experimental conditions.

**Figure 1:**
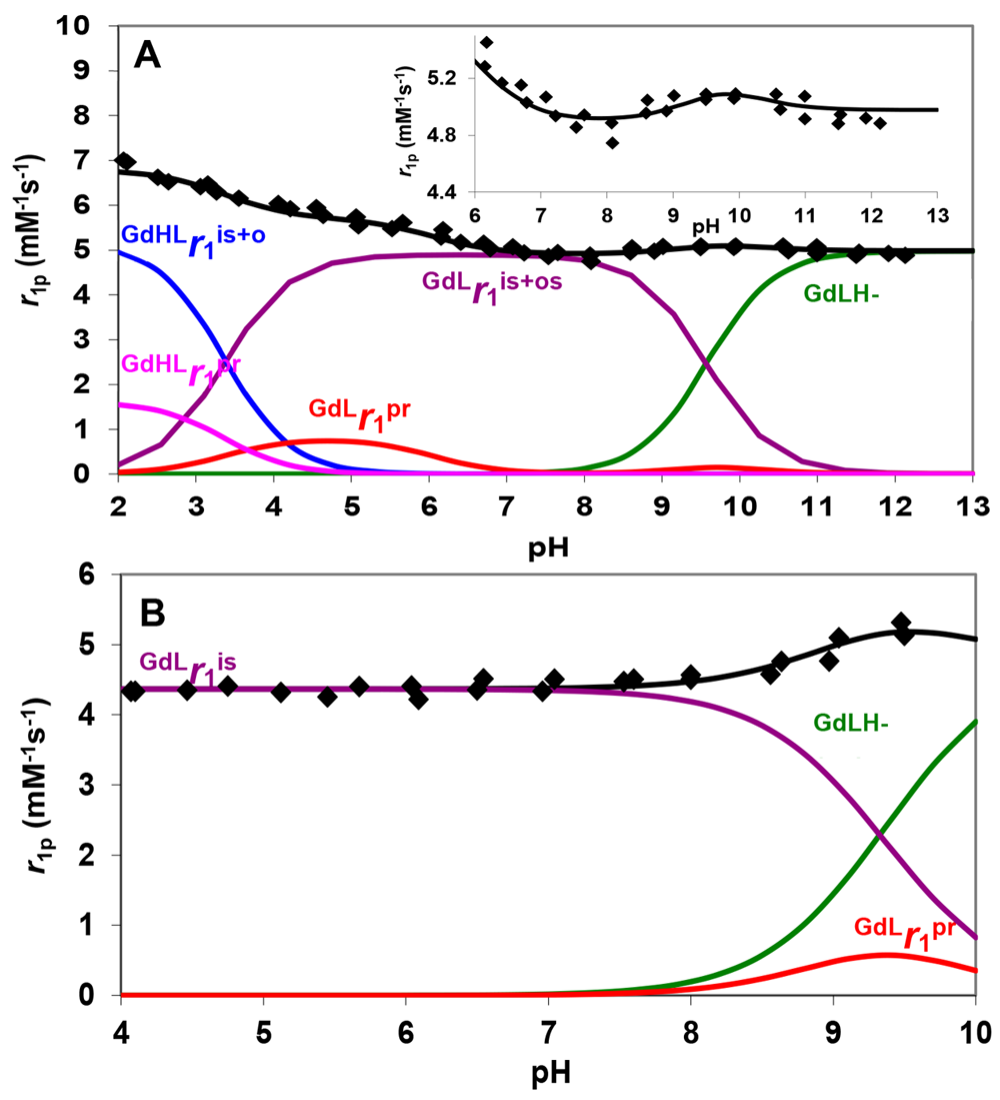
Relaxivity values (⧫) of GdL2 (A) and GdL1 (B). Symbols and solid lines represent experimental and calculated relaxivity values, respectively. Calculations were performed using eq 3 (20 MHz, 0.15 M NaCl, 298 K).

The relaxivity vs pH profiles of GdL1 and GdL2, measured at 21 MHz and 298 K in 0.15 M NaCl, are shown in Figure 1. At pH >3, the *r*_1_ values of GdL2 decrease with the increase of pH from 6.6 mM^–1^ s^–1^ to reach a plateau of 5.1 mM^–1^ s^–1^ around pH = 7.5. A further increase of the pH results in the slight increase of *r*_1_ reaching a maximum around pH = 9.5. Finally, at pH >9.5, another small decrease in *r*_1_ is observed. On the other hand, in the case of GdL1, the *r*_1_ values are independent from the pH in the range 4.0–7.5, whereas at pH >8.0, *r*_1_ increases up to 5.2 mM^–1^ s^–1^. The *r*_1_ values of GdL1 were measured in the pH range 4–10 in order to avoid the hydrolysis of the methyl ester. Since the carboxylate group of the 2-hydroxypropanoic pendant does not coordinate to the Gd^III^ ion, it can be assumed that the *r*_1_ increase in acidic and basic conditions for GdL2 and in basic conditions for GdL1 is due to acid- and/or base-catalyzed proton exchange between the −OH group of Gd^III^ complexes and the bulk. Then, the overall relaxivity, *r*_1_, is given by eq 1:
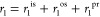
1where *r*_1_^is^, *r*_1_^os^, and *r*_1_^pr^ are the relaxivity components due to the inner and outersphere water molecules and the −OH group, respectively. *r*_1_^pr^ can be expressed as follows:^26−28^
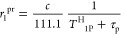
2

Here, *c* is the concentration of the complex, and *T*^H^_1P_ and τ_p_ are the longitudinal relaxation time and the lifetime of the −OH proton, respectively.

To explain the characteristic pH dependence of *r*_1_ (Figure 1), the species distribution of GdL1 and GdL2 as a function of pH must be considered (Table 1 and Table S1 and Figure S1). At pH >3, the deprotonation of the propanoic acid of the Gd(HL2) species results in the formation of [GdL2]^−^, which dominates in the pH range 5–8. On the other hand, the lack of the protonable side chain in L1 results in the dominance of the GdL1 species in the pH range 4–8. At pH >8, the Gd(L1)H_–1_ and Gd(L2)H_–1_ species were formed by deprotonation of the hydroxyl −OH group in the pendant arm. The unusual *r*_1_ vs pH dependence for GdL2 can be interpreted by considering the deprotonation of the complex at around pH 3–5 (Gd(HL2)) and 8–11 ([GdL2]^−^) and by the acid- and base-catalyzed proton exchange of the −OH proton. In the case of GdL1, which does not have the carboxylic proton, there is no acid-catalyzed proton exchange contribution; however, at pH >8, the base-catalyzed exchange of the −OH proton causes the increase of the *r*_1_ values as in GdHPDO3A.^5^

The inspection of Figure 1A and Figure S1 indicates that at pH <6 the molar ratio of the protonated Gd(HL2) species increases, and the protonated −COOH-assisted proton exchange between the coordinated OH group and the bulk becomes significant resulting in an increase in *r*_1_. This effect requires the fast proton exchange between the −COOH and the coordinated −OH groups in the Gd(HL2) species, followed by the rapid proton exchange with bulk water molecules. At pH <3, the general acid-catalyzed exchange of both −OH and −COOH protons with bulk water results in the further increase of *r*_1_. In GdL1 where the COOH proton is missing, this relaxation enhancement is not possible. On the other hand, similar phenomena were shown in the case of GdHPADO3A derivatives (HPADO3A, BzHPADO3A, and PipHPADO3A; Scheme 1), which are characterized by enhanced relaxivities due to the acid-catalyzed proton exchange between the labile amide protons and the −OH group.^7^ In addition, the *r*_1_ increase at pH >8 in both GdL1 and GdL2 can be interpreted by the additional contribution of OH^–^ ion-catalyzed proton exchange of the −OH group with bulk water. Then, at higher pH values, the deprotonation of the −OH group causes the decrease in the relaxivity values of GdL1 and GdL2 due to the loss of the exchangeable OH proton (Figure 1).

According to the proposed reaction mechanism, the rate of acid-catalyzed proton exchange of GdL2 and Gd(HL2) is *v*_H_ = *k*_H_[H_3_O^+^][GdL2] and *v*_H_ = *k*_H_[H_3_O^+^][Gd(HL2)], where the *k*_H_ rate constant characterizes the acid-catalyzed proton exchange processes of GdL2 and Gd(HL2) species. Because of the fast internal rearrangement, the alcoholic −OH and −COOH protons cannot be distinguished, and their lifetime is τ_p_ = (*k*_H_[H^+^])^−1^ in both cases. The rate of the base-catalyzed exchange between the alcoholic −OH proton of GdL1 and GdL2 and the bulk is *v*_OH_ = *k*_OH_[OH^–^][GdL] and τ_p_ = (*k*_OH_ [OH^–^])^−1^, where the rate constant *k*_OH_ characterizes the base-catalyzed proton exchange process for GdL1 and GdL2. Both acid- and base-catalyzed exchange mechanisms require the diffusion-controlled formation of an H-bonded complex and subsequently the rapid separation of the corresponding conjugate acid and base.^29^ By considering the proposed reaction mechanism, eq 2 can be rewritten as follows:
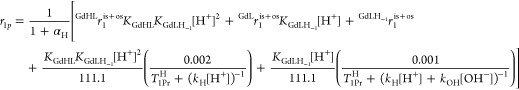
3where α_H_ = *K*_GdLH_–1__[H^+^] + *K*_GdHL_*K*_GdLH_–1__[H^+^]^2^, ^GdHL^*r*_1_^is+os^, ^GdL^*r*_1_^is+os^, and ^GdLH_–1_^*r*_1_^is+os^ are the sum of *r*_1_^is^ and *r*_1_^os^ for GdHL, GdL, and GdLH_–1_ species, respectively. Whereas for GdL2 the experimental data (Figure 1) were fitted to eq 3 (Table 2), in the case of GdL1, eq 3 was modified to account for the formation of GdL1 and Gd(L1)H_–1_ species and the base-catalyzed exchange of the −OH proton. Thus, the first and fourth terms and *k*_H_[H^+^] of the denominator in the last term in the brackets were not considered.

**Table 2:** Kinetic and Relaxation Parameters for the Proton Exchange Reactions of the Gd^III^ Complexes of L1, L2, HPADO3A, and HP-DO3A Ligands (20 MHz, 0.15 M NaCl, 298 K)

	^GdHL^*r*_1_^is+os^; mM^–1^ s^–1^	^GdL^*r*_1_^is+os^; mM^–1^ s^–1^	^GdLH_–1_^*r*_1_^is+os^; mM^–1^ s^–1^	*T*_1_^H^ × 10^6^; s	*k*_H_ × 10^–11^; M^–1^ s^–1^	*k*_OH_ × 10^–10^; M^–1^ s^–1^
GdL2	5.11 ± 0.08	4.85 ± 0.04	4.93 ± 0.03	8.5 ± 0.1	1.5 ± 0.2	0.17 ± 0.04
GdL1		4.40 ± 0.02	4.8 ± 0.1	5 ± 1		0.7 ± 0.1
GdHPADO3A^a^		4.57	4.32	5.6	2.1	
GdHPDO3A^a^		4.28	4.54	5.0		1.0

a Ref (7).

The comparison of the *r*_1_^is+os^ values (Table 2) indicates that the sums of the inner- and outer-contributions of GdL2, GdHL2, and GdL2H_–1_ species are very similar. However, *r*_1_^is+os^ values for GdL2 are about 0.5 mM^–1^ s^–1^ units higher than those of the corresponding GdL1, GdHPDO3A, and GdHPADO3A complexes, which might be explained by the presence of second sphere water molecules due to the hydrophilic nature of the extra carboxylate pendant. The H^+^-assisted exchange of the labile protons of GdL2 and GdHPADO3A are characterized by very similar *k*_H_ and *T*^H^_1P_ values (Table 2), confirming the analogous behavior of these complexes. The *k*_H_ rate constant is about an order of magnitude larger than the rate constant characterizing the diffusion-controlled acid- and base-catalyzed proton exchange processes due to the simultaneous double-site exchange mechanism as proposed in the case of the proton exchange processes of GdHPADO3A.^7^ Regarding the *k*_OH_ rate constant, the values for GdL1 and GdHPDO3A are similar and about 4 times higher than that of GdL2. By taking into account the factors influencing the proton exchange processes, the formation of an internal H-bond between the −OH proton and the deprotonated carboxylate group of the arm reduces the rate of the base-catalyzed intermolecular proton exchange process, due to the requirement to break the H-bond before exchanging the proton with the OH^–^ ions.

**Figure 2:**
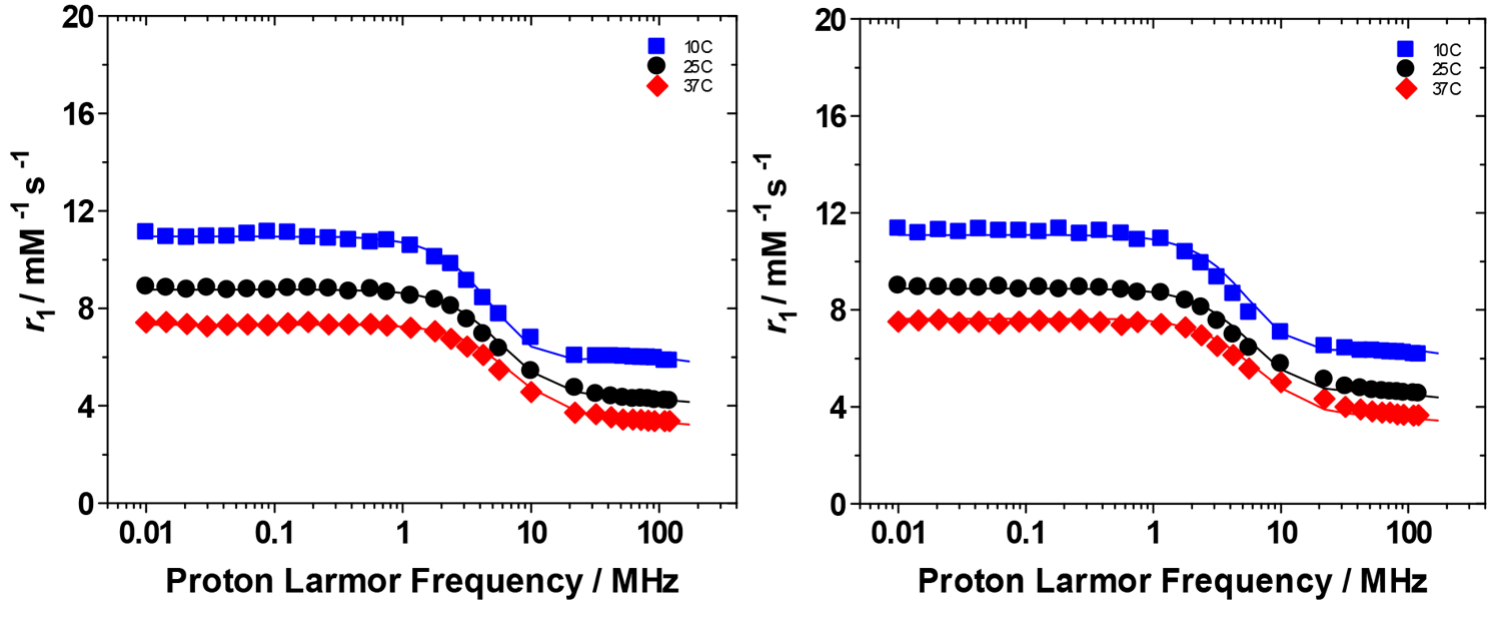
^1^H NMRD profiles acquired at pH 7.4 and 283 (blue ■), 298 (black ●), and 310 K (red ⧫) for aqueous solutions of GdL1 (left) and GdL2 (right). The solid lines represent the best-fitting results of the experimental data points.

The relaxometric characterization of the Gd complexes was carried out by recording their ^1^H nuclear magnetic relaxation dispersion (NMRD) profiles at 283, 298, and 310 K in the proton Larmor frequency range 0.01–120 MHz, corresponding to magnetic field strengths varying between 2.34 × 10^–4^ and 3 T (Figure 2). The shape of the NMRD profiles and their temperature dependence (*r*_1_ decreases with increasing temperature) reflect the general behavior of small Gd^III^ complexes, characterized by a constant *r*_1_ value at low fields, a dispersion around 4–6 MHz, and another region at high fields (>20 MHz) where *r*_1_ slightly decreases. The temperature dependence of *r*_1_ over the entire range of proton Larmor frequencies considered indicates that the coordinated water molecule is in fast exchange with the bulk, and thus, *r*_1_ is not limited by the water exchange rate but rather by the rotational motion. A least-squares fit of the profiles was carried out in terms of the established theory of paramagnetic relaxation expressed by the Solomon–Bloembergen–Morgan^30,31^ and Freed’s^32^ equations for the innersphere (IS) and outersphere (OS) proton relaxation mechanisms, respectively (Table 3). Because of the large number of parameters involved in the fitting procedure, some of them were fixed to known or reasonable values: The hydration number *q* was fixed to 1, and the distance between Gd^III^ and the protons of the bound water molecule, *r*, was fixed to 3.0 Å; the distance of closest approach, *a*, of the *outersphere* water molecules to Gd^III^ was set to 4.0 Å, and for the relative diffusion coefficient *D*, standard values of 1.7, 2.24, and 3.1 × 10^–5^ cm^2^ s^–1^ (283, 298, and 310 K) were used. Notably, the ester or carboxylic substituents in L1 and L2 are too far from the coordinated water to affect *r*_Gd–H_. The fit was performed using as adjustable parameters τ_R_ and the electronic relaxation parameters Δ^2^ (trace of the squared zero-field splitting, ZFS, tensor) and τ_V_ (correlation time for the modulation of the transient ZFS). The difference in relaxivity between GdL1 and GdL2 cannot account for the difference in τ_R_ since their molecular weight is very similar; therefore, we considered that the pendant propionate moiety of GdL2 allows for the presence of second sphere (SS) water molecules in H-bonding interaction with the carboxylate group at a distance from Gd^III^ sufficiently short (<4 Å) and with a residence time sufficiently long to be affected by the rotation. Notably, also in the case of GdHPADO3A, the amide group favors the H-bond interaction of second sphere water molecules that contribute to *r*_1_.^7^ Therefore, we analyzed the NMRD profiles for GdL2 also considering the SS contribution, expressed in terms of two additional parameters: the number *q*_SS_ of second sphere water molecules and their rotational correlation time, τ_R(SS)_. The average distance from the paramagnetic center was arbitrarily fixed at 3.5 Å, an intermediate value between those of water molecules in the inner (3.0 Å) and outer (4.0 Å) solvation shell.^33^ In addition, since GdL1 and GdL2 are structurally related to the recently reported GdHPADO3A-like complexes having a carbonyl group in place of the methyl group present in GdHPDO3A, *τ*_M_ was fixed to that reported for GdHPADO3A at pH 7 (20 ns).^7^ The electronic and reorientation parameters reported in Table 3 for GdL1 and GdL2 are in good agreement with those of other GdHPDO3A-like complexes. Finally, considering the contribution of two second sphere water molecules characterized by a rotational correlation time τ_R(SS)_ of 30 ps, the contribution of the SS molecules to the relaxivity of GdL2 is about 0.3 mM^–1^ s^–1^ at 20 MHz and 298 K almost equal to the Δ*r*_1_ between GdL1 and GdL2.

**Table 3:** Selected Parameters Obtained from the Analysis of the 1/*T*_1_ NMRD Profiles for GdL1 and GdL2 Compared to Other GdHPDO3A-like Complexes^a^

parameter	GdL1	GdL2	GdHPADO3A^7^	GdHPDO3A^24^^,^^b^	GdHPDO3MA^25^^,^^b^
^298^*r*_1-60-MHz_/mM^–1^ s^–1^	4.3 ± 0.1	4.6 ± 0.1	3.6	4.2	4.7
^310^*r*_1-60-MHz_/mM^–1^ s^–1^	3.4 ± 0.1	3.6 ± 0.1	2.9	3.2	3.6
*τ*_R_/ps	65 ± 3	68 ± 2	62	65	75
*Δ*^2^/10^19^ s^–2^	5.9 ± 0.3	8.5 ± 0.2	8.5	9.9; 1.5	3.0; 2.6
*τ*_v_/ps	14.6 ± 0.6	13.7 ± 0.5	14	8; 30	18; 25
*τ*_M_/ns	20^c^	20^c^	20	640; 8.5	64; 3
*τ*_R(SS)_^d^		30 ± 2	13		

a The following parameters were fixed during the fitting procedure: *q* = 1; *r*_Gd–H_ = 3.0 Å; *a* = 4.0 Å; *D*^298^ = 2.24 × 10^–5^ cm^2^ s^–1^; *A*/*ℏ* = −3.3 × 10^6^ rad s^–1^.

b Values for the SAP isomer are listed first; those for the TSAP isomer are listed second.

c τ_M_ value fixed to the value determined for GdHPADO3A.

d Determined for *q*_SS_ = 2 and *r*_SF_ = 3.5 Å.

It should be noted that Gd-DOTA-like complexes are typically characterized by the presence of two different coordination isomers defined by the same conformation of the macrocyclic ring but with a different orientation of the side arms (i.e., capped square–antiprismatic geometry, SAP, and capped twisted square antiprismatic geometry, TSAP).^1,2^ These isomers are characterized by significantly different rates of water exchange that, in the case of GdHPDO3A^24^ and GdHPDO3MA,^25^ were determined experimentally (Table 3). On the other hand, the analysis of the stereoisomers was not attempted in this work.

### X-ray Structure of the [Gd(L2)H_–1_(OH^–^)]^3–^ Complex

**Figure 3:**
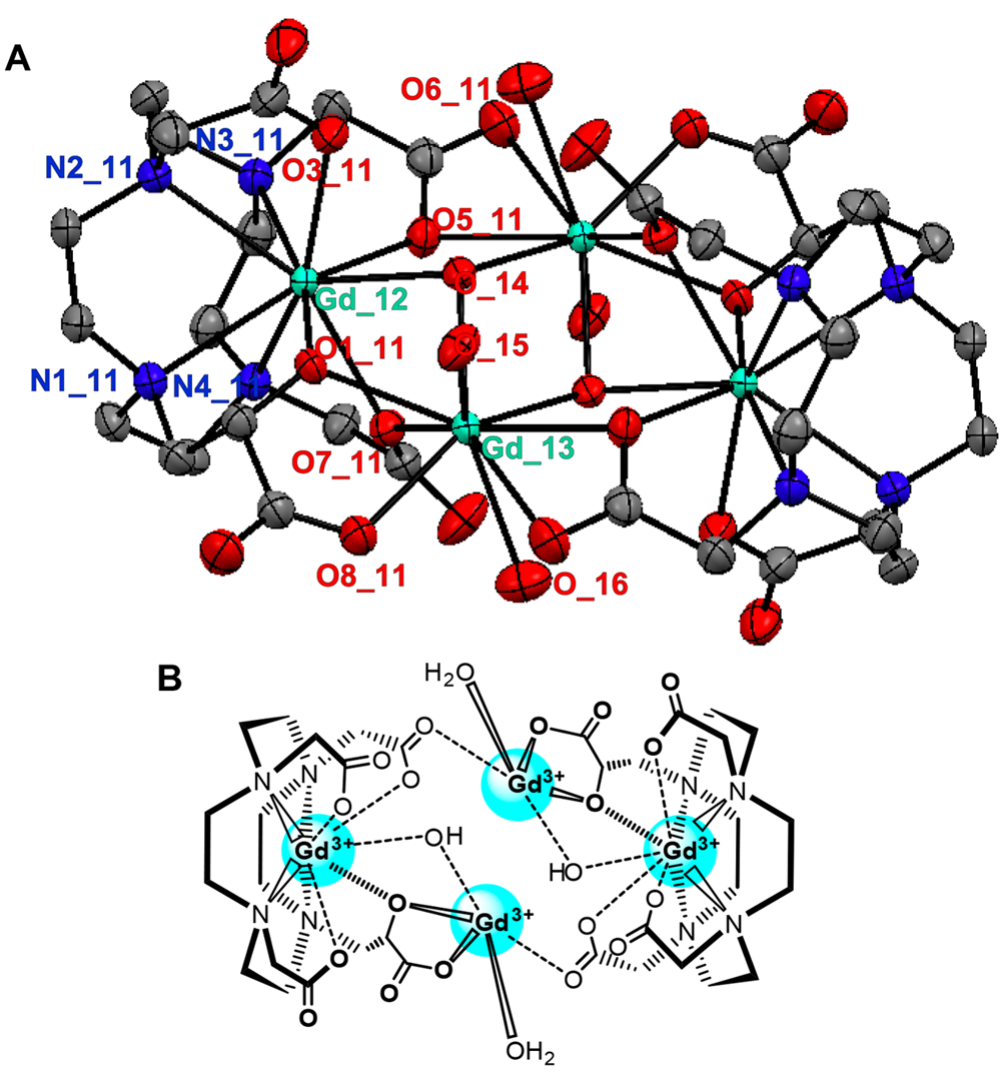
(A) View of the [Gd(L2)H_–1_(HO^–^)] complex present in the single crystal of {[(Gd(H_2_O)_2_)_2_[Gd(L2)H_–1_(HO^–^)]_2_} × 20H_2_O. Hydrogen atoms are omitted for clarity. Color code: Gd (green), O (red), N (blue), and C (gray). Selected bond distances (Å): Gd_12–N1_11 2.659(2), Gd_12–N2_11 2.662(2), Gd_12–N3_11 2.749(2), Gd_12–N4_11 2.703(2), Gd_12–O1_11 2.289(2), Gd_12–O3_11 2.406(2), Gd_12–O5_11 2.357(2), Gd_12–O7_11 2.444(2), Gd_12–O_14 2.461(1), and Gd_13–O_14 2.425(2). (B) Simplified chemical structure of the dimeric tetranuclear {[(Gd(H_2_O)_2_)_2_[Gd(L2)H_–1_(HO^–^)]_2_} complex.

To investigate in detail the structural properties of GdL2, single crystals of formula {[(Gd(H_2_O)_2_)_2_[Gd(L2)H_–1_(HO^–^)]_2_} × 20 H_2_O were grown at pH 9 with a slight excess of Gd(OH)_3_. The crystal structure was determined by X-ray diffraction studies, and a simplified structure of the mononuclear [Gd(L2)H_–1_(HO^–^)]^3–^ complex with selected bond distances is given in Figure 3. Other details regarding the structure of [Gd(L2)H_–1_(HO^–^)]^3–^ are reported in the Supporting Information.

Interestingly, the crystal structure reveals the presence of a dimer formed by two [Gd(H_2_O)_2_]^3+^ and two [Gd(L2)H_–1_(HO^–^)]^3–^ complexes forming a dimeric tetranuclear system (Figure 3 and Figures S5 and S6). A crystallographic inversion center lies on the dimeric [Gd(L2)H_–1_(OH^–^)]^3–^ barycenter; therefore, the crystallographic asymmetric unit (ASU) contains a single L2 moiety, and the complete molecule bears an equal population of enantiomers formed by the 2-hydroxypropionic arm of L2. Crystal packing voids are filled with water molecules tightly bound to the dimeric [Gd(L2)H_–1_(OH^–^)]^3–^ complex through a strong network of hydrogen bonds (Figures S6 and S7 and Table S4).

As shown from the chemical draw in Figure 3B, in the dimer, eight carboxylate-, two alkoxide-, and two hydroxide-oxygen atoms of the two [Gd(L2)H_–1_(HO^–^)]^3–^ complexes are coordinated to the two [Gd(H_2_O)_2_]^3+^ units. In addition, two hydroxide anions are in the bridge positions coordinating to both [Gd(H_2_O)_2_]^3+^ units. The coordination geometry around the Gd^III^ ion in the [Gd(H_2_O)_2_]^3+^ unit is a distorted tricapped trigonal prism with the capping positions occupied by a carboxylate oxygen of the 2-hydroxypropionic pendant (O8A_11), water oxygen (O_15), and the bridging hydroxide oxygen (O_14) atoms. The Gd^III^ ion is placed between two trigonal planes formed by carboxylate (O7_11), alkoxide (O1_11), and the other hydroxide (O_14) oxygen atoms and by two carboxylate (O5_11, O6_11) and water oxygen (O_16) atoms (Figure 3 and Figures S5 and S6). A charge balance suggests that an oxygen atom (O_14) is part of the hydroxide anion which is confirmed by the location of one apical hydrogen atom obtained from the electron density Fourier difference maps.

On the other hand, four nitrogen atoms, three carboxylates, and the alkoxide oxygen atom of L2 and one hydroxide oxygen atom in the capping position provide the coordination polyhedron around the Gd^III^ ion in [Gd(L2)H_–1_(HO^–^)]^3–^ (Figure 3 and Figures S5 and S6). The eight donor atoms of L2 encapsulate the central Gd^III^ ion between the four coplanar macrocyclic nitrogen atoms (N1_11, N2_11, N3_11, and N4_11) and the four coplanar oxygen atoms of three acetic and the 2-hydroxypropionic pendant arms (O3_11, O5_11, O7_11, and O1_11). The ninth capping coordination site of the Gd^III^ ion is occupied by the hydroxide anion (Gd_12–O_14: 2.461 Å). The torsion angles between the two square planes defined by the oxygen and nitrogen atoms are +8.9° and −8.9°. The coordination geometry around the Gd^III^ ion is a distorted monocapped twisted square antiprism (TSAP). The distance of the Gd^III^ ion from the O3_11–O5_11–O7_11–O1_11, and N1_11–N2_11–N3_11–N4_11 planes is 0.877 and 0.897 Å, respectively. The bond distances of Gd^III^ with the N and O donor atoms of the L2 ligand are in the ranges 2.66–2.75 Å and 2.29–2.44 Å, respectively.

The X-ray structure of [Gd(L2)H_–1_(HO^–^)]^3–^ is very similar to that of [Gd(HPDO3A)(H_2_O)]^20^ which has the same donor atoms in the nonadentate coordination sphere. However, in the two independent [Gd(HPDO3A)(H_2_O)] complexes, the torsion angles between the two square planes are 38° and −28° which are characteristic for capped square antiprismatic geometry (SAP) and capped twisted square antiprism (TSAP) stereoisomers, respectively. The distances of the Gd^III^ ion from the planes of nitrogen and oxygen atoms are 1.61 and 0.75 Å for the SAP and 1.68 and 0.78 Å for TSAP stereoisomers, respectively. The distances of Gd^III^–OH_2_ and Gd^III^–OH bonds are 2.51 and 2.32 Å for SAP and 2.50 and 2.35 Å for the TSAP isomer, respectively. The Gd^III^–N and Gd^III^–O distances are 2.64–2.65 and 2.31–2.38 Å, respectively.

According to the X-ray structure of the tetranuclear dimer [(Gd(H_2_O)_2_)(Gd(L2)H_–1_(HO^–^)] (Figure 3), each [Gd(H_2_O)_2_]^3+^ center is coordinated by the alkoxide and the carboxylate oxygen atoms of the 2-hydroxypropanoic arm of the [Gd(L2)H_–1_(HO^–^)]^3–^ units (Figure 3 and Figures S5 and S6). Assuming the presence of the protonated −OH group, we can hypothesize the formation of a five-membered ring with the proton of the hydroxyl −OH group bridging via an H bond with the carboxylate O^–^ donor atom. This is a confirmation of the involvement of these donor atoms in the intramolecular catalysis of the proton exchange of the −OH group with the water protons.

## Conclusions

The insertion of peripheral functional groups on the hydroxypropyl pendant arm on the HPDO3A ligand was shown to modulate the relaxivity of the Gd^III^ complexes without strongly affecting both thermodynamic stability and kinetic inertness (log *K*_GdL_ = 19.26, *t*_1/2_ = 2.14 × 10^7^ h, pH = 7.4, 0.15 M NaCl, 25 °C). The presence of an ester and carboxylic acid groups in place of the methyl group of HPDO3A in the new ligand synthesized (L1 and L2) demonstrated the importance of the proton on the carboxylic acid to allow intra- and intermolecular proton exchange with the coordinated hydroxyl group. The acid-catalyzed proton exchange causes a ca. 30% relaxivity increase at pH <6 in the case of GdL2, highlighting the effectiveness of the carboxylic group in catalyzing the proton movement through the formation of a five-membered ring with the Gd^III^-coordinated hydroxyl group. On the other hand, the base-catalyzed proton exchange has a minor influence on the relaxivity at pH >8 on both GdL1 and GdL2. The X-ray crystal structure of the tetranuclear dimer [(Gd(H_2_O)_2_)(Gd(L2)H_–1_(HO^–^)_2_] featured the presence of a dimer formed by two [Gd(H_2_O)_2_]^3+^ and two [Gd(L2)H_–1_(HO^–^)]^3–^ complexes with alkoxide and hydroxide ions bridging two pairs of Gd^III^ ions. Interestingly, the alkoxide and the carboxylate oxygen atoms of the 2-hydroxypropionic arm of the [Gd(L2)H_–1_(HO^–^)]^3–^ units form a five-membered ring with each [Gd(H_2_O)_2_]^3+^ center, similarly to what is hypothesized in the proton exchange mechanism where the proton moves from the OH to the carboxylate. Finally, although in the present example the relaxivity at physiological pH was not enhanced, we would like to remark that a proper selection of a secondary, noncoordinating functional group would not modify the stability of the Gd^III^ complex but may strongly influence the relaxivity of a Gd^III^-based agent through intra- or intermolecular proton exchange processes.

## Experimental Section

### Materials and Methods

All chemicals were purchased from Sigma-Aldrich or Alfa Aesar unless otherwise stated and were used without further purification. The ^1^H and ^13^C NMR spectra were recorded using a Bruker Avance III 500 MHz (11.4 T) spectrometer equipped with 5 mm PABBO probes and a BVT-3000 temperature control unit. Chemical shifts are reported relative to TMS and were referenced using the residual proton solvent resonances. Electrospray ionization mass spectra (ESI MS) were recorded using an SQD 3100 mass detector (Waters), operating in positive or negative ion mode, with 1% v/v formic acid in methanol as the carrier solvent.

HPLC analyses and mass spectra were performed on a Waters HPLC-MS system equipped with Waters 1525 binary pumps. Analytical measurements were carried out on a Waters XBridge-Phenyl column (5 μm 4.6 × 150 mm) using the following method (Method A): A = H_2_O/0.1% TFA; B = MeOH; flow = 1 mL/min; 0–2 min = 99% A; 2–15 min = from 99% A to 100% B; 15–19 min = 100% B; 19–20 min = from 100% B to 99% A.

Semipreparative HPLC purifications were performed on a Waters XBridge-Phenyl Prep OBD column (5 μm, 19 × 100 mm) using the following method (Method B): A = H_2_O/0.1% TFA; B = MeOH; flow = 20 mL/min; 0–3 min = 99% A; 3–7 min = from 99% A to 100% B; 7–8 min = 100% B; 8–9 min = from 100% B to 99% A.

#### Tri-*tert*-butyl(*R*)2,2′,2″-(10-(2-hydroxy-3-methoxy-3-oxopropyl)-1,4,7,10-tetraazacyclododecane-1,4,7-triyl) Triacetate (L1(O*t*Bu)_3_)

A solution of DO3A(*t*Bu)_3_ (200 mg, 0.4 mmol) and (*R*)-methylglicidate (172 μl, 4 mmol) in *t*-BuOH (4 mL) was stirred for 16 h under reflux. The solvent was removed in vacuo, and then, the reaction mixture was purified by silica gel chromatography (90:10 CH_2_Cl_2_:MeOH, *R*_f_ = 0.45) to afford compound (L1(O*t*Bu)_3_) (192 mg, 0.31 mmol, yield 78%). ^1^H NMR (CDCl_3_, 500 MHz): δ 1.46 (s, 27H, −NCH_2_COOC(C*H*_3_)_3_), 3.97–2.97 (m, 27H, macrocycle, −NC*H*_2_COOC(CH_3_)_3_, −OC*H*_3_, −NC*H*_2_CH(OH)−), 4.68 (m, 1H, −NCH_2_C*H*(OH)−). ^13^C NMR (CDCl_3_, 125 MHz): δ 171.1 (−CH(OH)*C*OOCH_3_), 166.5 (−NCH_2_*C*OOC(CH_3_)_3_), 83.8–83.1 (−*C*(CH_3_)_3_), 65.5 (−NCH_2_*C*H(OH)−), 54.9 (−N*C*H_2_CH(OH)−), 54.4 (−N*C*H_2_COOC(CH_3_)_3_), 52.9 (−O*C*H_3_), 48.1–51.0 (macrocycle), 27.9 (−C(*C*H_3_)_3_). ESI-MS (*m*/*z*): [M + H^+^] calcd for C_30_H_57_N_4_O_9_, 617.81; found, 617.7.

#### (*R*)2,2′,2″-(10-(2-Hydroxy-3-methoxy-3-oxopropyl)-1,4,7,10-tetraazacyclododecane-1,4,7-triyl) Triacetic Acid (L1)

L1(O*t*Bu)_3_ (96 mg, 0.16 mmol) was dissolved in DCM:TFA (1:1/v:v) (8 mL) and stirred at room temperature (rt), for 16 h. After the evaporation of the solvent in vacuo, the mixture was purified by semipreparative HPLC-MS with Method B reported in the Materials and Methods section. After HPLC-MS purification, the ligand L1 was dissolved in HCl 1 M (1 mL) and evaporated in vacuo. This operation was repeated twice, and finally, the aqueous solution was freeze-dried to obtain L1 as a HCl salt in 75% yield (62 mg, 0.12 mmol). ^1^H NMR (D_2_O, 500 MHz): δ 4.08 (m, −NCH_2_C*H*(OH)–, −NC*H*_2_CH(OH)–, 3H), 3.79–3.19 (m, macrocycle, −OC*H*_3_, −NC*H*_2_COOH, 25H). ^13^C NMR (D_2_O, 125 MHz): δ 172.8 (−CH(OH)*C*OOCH_3_), 169.5 ((−*C*OOH), 65.8 (−NCH_2_*C*H(OH)−), 54.7 (−N*C*H_2_CH(OH)−), 54.3 (−N*C*H_2_COOH), 53.27 (−O*C*H_3_), 51.4–48.9 (macrocycle). Analytical HPLC-MS (Method A): *t*_r_ = 10.24 min. ESI-MS (*m*/*z*): [M + H^+^] calcd for C_18_H_33_N_4_O_9_, 449.22; found, 449.5.

#### (*R*)2-Hydroxy-3-(4,7,10-tris(2-(*tert*-butoxy)-2-oxoethyl)-1,4,7,10-tetraazacyclododecan-1-yl) Propanoic Acid (L2(O*t*Bu)_3_)

L1(O*t*Bu)_3_ (96 mg, 0.16 mmol) was dissolved in an aqueous solution of LiOH (2 mL, 2M) and methanol (2 mL), and the resulting solution was stirred for 4 h at 50 °C. The reaction mixture was brought to pH 5; the methanol was removed by rotary evaporation, and the aqueous residue was extracted with DCM twice (4 mL). The organic phase was washed with brine, dried over anhydrous Na_2_SO_4_, and evaporated to yield L2(*t*Bu)_3_ in 66% yield (63 mg, 0.10 mmol). ^1^H NMR (CDCl_3_, 500 MHz): δ 1.44 (s, 27H, −NCH_2_COOC(C*H*_3_)_3_), 3.97–2.80 (m, macrocycle, −N*CH*_2_COOC(CH_3_)_3_, −NC*H*_2_CH(OH)–, 24H), 4.64 (m, 1H, −NCH_2_C*H*(OH)−). ^13^C NMR (CDCl_3_, 125 MHz): δ 170.6 (−CH(OH)*C*OOH), 166.2 (−NCH_2_*C*OOC(CH_3_)_3_), 84.9 (−*C*(CH_3_)_3_), 65.4 (−NCH_2_*C*H(OH)−), 54.9 (−N*C*H_2_CH(OH)−), 54.3 (−N*C*H_2_COO C(CH_3_)_3_), 50.9–49.3 (macrocycle), 27.9 (−C(*C*H_3_)_3_). ESI-MS (*m*/*z*): [M + H^+^] calcd for C_29_H_55_N_4_O_9_, 603.39; found, 603.1.

#### (*R*)2,2′,2″-(10-(2-Carboxy-2-hydroxyethyl)-1,4,7,10-tetraazacyclododecane-1,4,7-triyl) Triacetic Acid (L2)

L2(O*t*Bu)_3_ (63 mg, 0.10 mmol) was dissolved in DCM:TFA (1:1/v:v) (8 mL) and stirred at rt for 16 h. After the evaporation of the solvent in vacuo, the mixture was purified in semipreparative HPLC-MS with Method B reported in the Materials and Methods section. After HPLC-MS purification, the ligand L2 was dissolved in HCl 1 M (1 mL) and evaporated in vacuo. This operation was repeated twice, and finally, the aqueous solution was freeze-dried to obtain L2 as a HCl salt in 60% yield (30 mg, 0.06 mmol). ^1^H NMR (D_2_O, 500 MHz): δ 3.87 (m, −NCH_2_C*H*(OH)–, −N*CH*_2_COOH, 7H), 3.43–3.09 (m, macrocycle, −NC*H*_2_CH(OH)–, 18H). ^13^C NMR (D_2_O, 125 MHz): δ 175.2 (NCH_2_*C*OOH), 174.7 ((−CH(OH)*C*OOH), 66.1 (−NCH_2_*C*H(OH)−), 55.1 (−N*C*H_2_CH(OH)−), 53.5 (−N*C*H_2_COOH), 50.8–49.00 (macrocycle). Analytical HPLC-MS (Method A): *t*_r_ = 5.48 min. ESI-MS (*m*/*z*): [M + H^+^] calcd for C_17_H_30_N_4_O_9_, 435.2; found, 435.5.

### Preparation of Gd^III^ Complexes

L1 and L2 ligands (L1, 31 mg, 0.06 mmol; L2, 15 mg, 0.03 mmol) were dissolved in H_2_O (1 mL), and 1 equiv of GdCl_3_ dissolved in H_2_O (0.2 mL) was added. The pH was brought to 7 by small additions of NaOH 0.1 M, and the resulting solution was kept at room temperature overnight and finally lyophilized to obtain the final complexes.

#### GdL1

Analytical HPLC-MS (Method A): *t*_r_ = 9.4 min. ESI-MS (*m*/*z*): [M + H]^+^ calcd for C_18_H_29_GdN_4_O_9_, 604.12; found, 604.44.

#### GdL2

Analytical HPLC-MS (Method A): *t*_r_ = 8.8 min. ESI-MS (*m*/*z*): [M + H]^+^ calcd for C_17_H_31_GdN_4_O_9_, 590.13; found, 590.49. Single crystals suitable for X-ray diffraction studies were grown by the slow evaporation of water from a solution of GdL2 at pH = 9.0 in the presence of excess Gd(OH)_3_.

### Equilibrium Measurements

#### Materials

The chemicals used for the experiments were of the highest analytical grade. The concentrations of the CaCl_2_, ZnCl_2_, CuCl_2_, and GdCl_3_ solutions were determined by complexometric titration with standardized Na_2_H_2_EDTA and xylenol orange (ZnCl_2_, and GdCl_3_), murexid (CuCl_2_), and Patton and Reeder (CaCl_2_) as indicators. The concentration of the H_3_L1 and H_4_L2 was determined by pH potentiometric titration in the presence and absence of a large (40-fold) excess of CaCl_2_. The pH potentiometric titrations were performed with standardized 0.2 M NaOH.

#### Equilibrium Measurements

The stability and protonation constants of Ca^2+^, Zn^2+^, and Cu^2+^ complexes formed with the L1 ligand were determined by pH potentiometric titration. The metal-to-ligand concentration ratio was 1:1 (the concentration of the ligand was generally 0.002 M). The protonation constants of the GdL1 and GdL2 complexes were determined using pH potentiometry by titrating the preprepared complexes from pH = 3.0 to pH = 12 for GdL2 and from pH = 4.0 to pH = 12 for GdL1 with 0.2 M NaOH. The stability constants of GdL2 were determined by the “out-of-cell” technique because of the slow formation reaction. The pH range of the complexation equilibria and the time needed to reach the equilibria were determined by relaxometry for the formation of GdL2. Eight Gd^3+^–L2 samples were prepared, which had pH values in the range 2.5–4.0 at equilibrium ([Gd^3+^] = [L2] = 0.002 M). The samples were kept at 25 °C for 10 weeks to reach equilibrium. For the calculation of the stability constants of GdL2, besides the protonation constants of ligands, the stability constants of the diprotonated *Gd(H_2_L2) out-of-cage complexes (considered as intermediates) were also used as fixed values, which were calculated from the pH potentiometric titration curve of the Gd^3+^–L2 system obtained in the pH range 1.7–4.0.

For the pH measurements and titrations, the Metrohm 888 Titrando titration workstation Metrohm-6.0234.110 combined electrode was used. Equilibrium measurements were carried out at a constant ionic strength (0.15 M NaCl) in 6 mL samples at 25 °C. The solutions were stirred, and N_2_ was bubbled through them. The titrations were made in the pH range 1.7–12.0. KH-phthalate (pH = 4.005) and borax (pH = 9.177) buffers were used to calibrate the pH meter. For the calculation of [H^+^] from the measured pH values, the method proposed by Irving et al. was used as follows.^34^ A 0.01 M HCl solution was titrated with standardized NaOH solution at 0.15 M NaCl ionic strength. The differences (*A*) between the measured (pH_read_) and calculated (−log[H^+^]) pH values were used to obtain the equilibrium H^+^ concentration from the pH values measured in the titration experiments (*A* = 0.024). For the equilibrium calculations, the stoichiometric water ionic product (p*K*_w_) was also needed to calculate [OH^–^] values under basic conditions. The *V*_NaOH_–pH_read_ data pairs of the HCl–NaOH titration obtained in the pH range 10.5–12.0 were used to calculate the p*K*_w_ value (p*K*_w_ = 13.79).

The stability constants of CuL2 were determined by spectrophotometry studying the Cu^2+^–L2 systems at the absorption band of Cu^2+^ complexes at [H^+^] = 0.01–1.0 M in the wavelength range 210–350 nm. The concentrations of Cu^2+^ and L2 were 0.72 mM. The H^+^ concentration in the samples was adjusted with the addition of calculated amounts of 3 M HCl. (*I* = [Na^+^] + [H^+^] = 0.15, [H^+^] ≤ 0.15 M). The samples were kept at 25 °C for 1 week. The absorbance values of the samples were determined at 11 wavelengths (260, 269, 278, 287, 296, 305, 314, 323, 332, 341, and 350 nm). For the calculations of the stability and protonation constants of the CuL1, the molar absorptivities of CuCl_2_, CuL2, Cu(HL2), Cu(H_2_L2), and Cu(H_3_L2) were determined by recording the spectra of 1.0 × 10^–3^, 3.0 × 10^–4^, 6.0 × 10^–4^, and 9.0 × 10^–4^ M solutions of CuCl_2_ and CuL2 in the pH range 1.7–7.5. The pH was adjusted by stepwise addition of concentrated NaOH or HCl solutions. The spectrophotometric measurements were made with the use of a PerkinElmer Lambda 365 UV–vis spectrophotometer at 25 °C, using 1.0 cm cells. The protonation and stability constants were calculated with the PSEQUAD program.^35^

### Kinetic Studies

The kinetic inertness of the GdL2 was characterized by the rates of the dissociation reactions taking place in 0.01–1.0 M HCl solution. The dissociation reactions of the GdL2 were followed by measuring the longitudinal relaxation time of H_2_O protons (*T*_1_) with a Stelar relaxometer connected to a Bruker WP80 NMR electromagnet adapted to variable-field measurements (15–80 MHz proton Larmor frequency). The temperature was controlled with a Stelar VTC-91 airflow heater equipped with a calibrated copper–constantan thermocouple (uncertainty of ±0.1 °C). Measures were carried out at 21 MHz and 25 °C. The longitudinal relaxation time (*T*_1_) was measured with the “inversion recovery” method (180° – τ – 90°) by using 16 different τ values with a typical 90° pulse width of 6.5 μs, 4 scans. The measurements were performed with a 1 mM solution of the GdL2 complex. The relaxivity values were given as *r*_1_ = 1/*T*_1p_ + 1/*T*_1w_ where *T*_1p_ and *T*_1w_ are the relaxation times of the bulk water protons in the presence and absence of GdL2. The pseudo-first-order rate constants (*k*_d_) were calculated by fitting the relaxation rate (*r*_1_ = 1/*T*_1p_) data to eq 4.
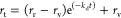
4where *r*_r_ and *r*_v_ are the relaxivity values of the reactants and the product (Gd^3+^: *r*_1p_ = 12.85 (2) mM^–1^ s^–1^, 21 MHz, 25 °C) and *r*_t_ is the measured relaxivity at reaction time *t*. The temperature was maintained at 25 °C, and the ionic strength of the solutions was kept constant at [H^+^] ≤ 0.15 M, [HCl] + [NaCl] = 0.15 M. The calculation of the kinetic parameters was performed by the fitting of the absorbance–time and relaxation rate–time data pairs with the Micromath Scientist computer program (version 2.0, Salt Lake City, UT).

### Relaxometric Measurements

Proton relaxation measurements (1/*T*_1_) and the resulting 1/*T*_1_ NMRD profiles were measured on a fast-field cycling (FFC) Stelar SmarTracer relaxometer over a continuum of magnetic field strengths from 0.000 24 to 0.25 T (corresponding to 0.01–10 MHz proton Larmor frequencies). The relaxometer operates under computer control with an absolute uncertainty in 1/*T*_1_ of ±1%. A precise control of the temperature was operated during the measurements by means of a Stelar VTC-91 airflow heater equipped with a calibrated copper constantan thermocouple (uncertainty of ±0.1 °C). Furthermore, the real temperature inside the probe head was additionally monitored by a Fluke 52 k/j digital thermometer (Fluke, Zürich, Switzerland). The gadolinium concentration was determined by measuring the bulk magnetic susceptibility shifts of the *t*-BuOH ^1^H NMR signal. Additional data in the 20–120 MHz frequency range were obtained with a high field relaxometer (Stelar) equipped with the HTS-110 3T metrology cryogen-free superconducting magnet. The data were collected using the standard inversion recovery sequence (20 experiments, 2 scans) with a typical 90° pulse width of 3.5 ms, and the reproducibility of the data was within ±0.5%. *r*_1_ values as a function of the pH were measured in nondeuterated aqueous solutions at 21 MHz on a Stelar relaxometer connected to a Bruker WP80 NMR electromagnet adapted to variable-field measurements (15–80 MHz proton Larmor frequency). The longitudinal relaxation time (*T*_1_) was measured with the “inversion recovery” method (180° – τ – 90°) by using 16 different τ values with typical 90° pulse width of 6.5 μs, 4 scans. The measurements were performed with a 1 mM solution of GdL1 and GdL2 complexes in the presence of 0.15 M NaCl ionic strength. The relaxivity values were given as *r*_1_ = 1/*T*_1p_ + 1/*T*_1w_ where *T*_1p_ and *T*_1w_ are the relaxation times of the bulk water protons in the presence and absence of the Gd^III^ complex. The pH-dependent relaxivity measurements of GdL1 and GdL2 complexes were carried out by direct titration of the samples (GdL2, 3.0 < pH < 12.5; GdL1, 4 < pH < 10). The pH was adjusted by the stepwise addition of concentrated NaOH or HCl solution. Calculations were performed with the computer program Micromath Scientist, version 2.0 (Salt Lake City, UT).

### X-ray Diffraction Studies

Data collections were performed at the X-ray diffraction beamline (XRD1) of the Elettra Synchrotron, Trieste, Italy.^36^ The crystals were dipped in NHV oil (Jena Bioscience, Jena, Germany) and mounted on the goniometer head with kapton loops (MiTeGen, Ithaca, NY). Complete data sets were collected at 100 K (nitrogen stream supplied through an Oxford Cryostream 700–Oxford Cryosystems Ltd., Oxford, United Kingdom) through the rotating crystal method. Data were acquired using a monochromatic wavelength of 0.700 Å, on a Pilatus 2 M hybrid-pixel area detector (DECTRIS Ltd., Baden-Daettwil, Switzerland). The diffraction data were indexed and integrated using XDS.^37^ X-ray data of the single crystals with the formula {[(Gd(H_2_O)_2_)_2_[Gd(L2)H_–1_(HO^–^)]_2_} × 20H_2_O were merged, scaled, and corrected for absorption using SADABS code.^38^ A triclinic crystal form has been found, and complete data sets have been obtained combining data from 2 or 3 φ scans, collected at different orientations from the same crystal. The structures were solved by the dual space algorithm implemented in the SHELXT code.^39^ Fourier analysis and refinement were performed by the full-matrix least-squares methods based on F^2^ implemented in SHELXL (Version 2018/3).^40^ The Coot program has been used for modeling.^41^ Anisotropic thermal motion refinement has been applied to all atoms. Hydrogen atoms were included at calculated positions with isotropic *U*_factors_ = 1.2*U*_eq_ or *U*_factors_ = 1.5*U*_eq_ for methyl and hydroxyl groups (*U*_eq_ being the equivalent isotropic thermal factor of the bonded nonhydrogen atom). Pictures were prepared using Ortep3,^42^ CCDC Mercury,^43^ and Pymol^44^ software. Essential crystal and refinement data are summarized in Table S2. Further crystallographic data are shown in the Supporting Information and deposited in the Cambridge Crystallographic Data Centre under CCDC 2087813.

## Supporting Information Available

Protonation and complexation equilibria of ligand and complexes; kinetic studies for GdL2; X-ray diffraction studies of the [Gd(L2)H_–1_(OH^–^)]^3–^ complex; and ^1^H and ^13^C NMR spectra of protected and deprotected ligands (PDF)

## Accession Codes

CCDC 2087813 contains the supplementary crystallographic data for this paper. These data can be obtained free of charge via www.ccdc.cam.ac.uk/data_request/cif, or by emailing data_request@ccdc.cam.ac.uk, or by contacting The Cambridge Crystallographic Data Centre, 12 Union Road, Cambridge CB2 1EZ, UK; fax: +44 1223 336033.
