# Supplementary material for: Underlining the Importance of Peripheral Protic Functional Groups to Enhance the Proton Exchange of Gd-Based MRI Contrast Agents
Source: Inorg Chem. 2021 Aug 13;60(17):13626–36. doi: 10.1021/acs.inorgchem.1c01927 (PMC8769378; doi:10.1021/acs.inorgchem.1c01927)
Supplement: Supplementary file 1 — ic1c01927_si_001.pdf [file ic1c01927_si_001.pdf]

Electronic Supplementary Information

for

Underlining the importance of peripheral protic functional groups to enhance the proton exchange of Gd-based MRI contrast agents.

Mariangela Boccalon,[a] Loredana Leone,[b] Giuseppe Marino,[a] Nicola Demitri,[c] Zsolt Baranyai,[a] Lorenzo Tei[b]**

[a] Bracco Research Centre, Bracco Imaging S.p.A. Via Ribes 5, 10010, Colleretto Giacosa, Italy.

[b] Dipartimento di Scienze e Innovazione Tecnologica, Università del Piemonte Orientale "A. Avogadro", Viale T. Michel 11, 15121 Alessandria, Italy.

[c] Elettra–Sincrotrone Trieste, S.S. 14 Km 163.5 in Area Science Park, 34149 Basovizza – Trieste, Italy.

CONTENT:

1. Protonation and complexation equilibria of L1 and L2 pag. 2

2. Kinetic inertness of GdL2 pag. 7

3. X-ray diffraction studies of $[Gd(L2)H_{-1}(OH^-)]^{3-}$ complex pag. 9

4. ^1H and ^{13}C NMR spectra of protected and deprotected ligands pag. 16

5. References pag. 23

1. Protonation and complexation equilibria of L1 and L2:

The protonation constants of H_4L2, defined by Eq. (S1), were determined by pH-potentiometry.

$$K_i^H = \frac{[H_iL]}{[H_{i-1}L][H^+]} \qquad (S1)$$

where i=1, 2…6. The $\log K_i^H$ values of L2 obtained by pH-potentiometry are listed and compared with those of H_3HPADO3A, H_3HPDO3A, H_3BT-DO3A and H_4DOTA (Scheme S1) in Table S1. Standard deviations (3σ) are shown in parentheses. By taking into account the protonation sequence of the macrocyclic DOTA-like ligands determined by spectroscopy and potentiometry methods,[1-3] it is assumed that the first and second protonation of L2 occur at two opposite ring nitrogen atoms, whereas the third protonation process takes place at one of the carboxylate groups attached to the non-protonated ring nitrogen atoms. Fourth proton is localized on the carboxylate group of the 2-hydroxypropanoic side chain, whereas further protonation of L2 occurs at the non-protonated carboxylate pendant arms. Comparison of the protonation constants (Table S1) reveals that the $\log K_1^H$ and $\log K_2^H$ values of L2, HPADO3A and BT-DO3A are comparable and significantly lower than that of HPDO3A and DOTA. In fact, equilibrium data characterizing the protonation of L1, L2, HPADO3A and BT-DO3A ligands were measured in the presence of 0.15 M and 0.1 M NaCl ionic strength, whereas those of HPDO3A and DOTA complexes were obtained in 0.1 M Me_4NCl and 0.1 M KCl solution, respectively. It is well known that DOTA derivatives form complexes with Na^+ ion ($\log K_{Na(DOTA)}$=4.38; $\log K_{Na(BT\text{-}DO3A)}$=2.32).[4,5], which are clearly responsible for the lower $\log K_1^H$ and $\log K_2^H$ values of L2, HPADO3A and BT-DO3A ligands.

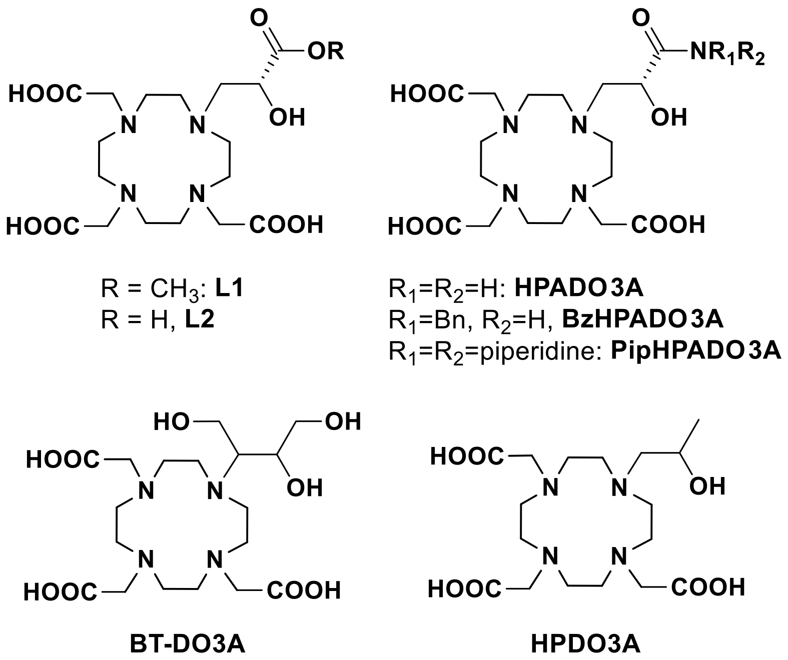

Scheme S1. Macrocyclic ligands discussed in the present work.

The stability and protonation constants of Ca^{II}-, Zn^{II}-, Cu^{II}- and Gd^{III}-complexes of L2, expressed by Eqs. (S2) and (S3), were determined by pH-potentiometry and spectrophotometry at 25 ºC in 0.15 M NaCl solution.

$$K_{ML} = \frac{[ML]}{[M][L]} \tag{S2}$$

$$K_{MH_iL} = \frac{[MH_iL]}{[MH_{i-1}L][H^+]} \tag{S3}$$

where i=0, 1, 2, 3. The K_{ML} and K_{MHiL} values of Ca^{II}-, Zn^{II}- and Cu^{II}-L2 complexes were calculated from the pH-potentiometric titration data obtained at 1:1 metal to ligand concentration ratios. In the calculations, the best fitting of the mL NaOH – pH data was obtained by assuming the formation of ML, MHL, MH_2L and MH_3L species. The stability constant of GdL2 was calculated from the equilibrium data obtained by the "out-of-cell" technique due to the slow complexation reaction.
The formation of lanthanide(III)-complexes with DOTA and DOTA derivatives takes place via the diprotonated "out-of-cage" intermediate species (e.g. *Ln(H_2DOTA)), in which the Ln^{III}-ion is coordinated by the carboxylate groups, whereas two opposite ring nitrogens are protonated.[6-10] Tipically, the formation of the "in cage" $Ln(DOTA)^-$ and LnDOTA-like complexes occurs by the slow deprotonation of the ring nitrogens, which is followed by the penetration of the Ln^{III}-ion into the N and O donors coordination cage of the ligand. In order to calculate reliable equilibrium constants, the presence of the $Ln(H_2DOTA)^+$ intermediate, the free Ln^{3+} ion and the final $Ln(DOTA)^-$ complex should be considered in the equilibrium Ln^{III} - DOTA system.[11,12] The formation and stability constant of the *Ln(H_2L) intermediate is expressed by Eqs (S4) and (S5).

$$Ln^{3+} \quad + \quad H_2L \quad \rightleftharpoons \quad *Ln(H_2L) \tag{S4}$$

$$^{*}K_{Ln(H_2L)} = \frac{[Ln(H_2L)]}{[Ln^{3+}][H_2L]} \tag{S5}$$

Since this out-of-cage intermediate is formed rapidly, the $*K_{Gd(H2L)}$ stability constant of the intermediate *Gd(H_2L2) was calculated from the equilibrium data obtained by the direct pH-potentiometric titration of Gd^{3+} - L2 systems at 1:1 metal to ligand concentration ratios in the pH range 1.7 – 4.0. On the other hand, the stability constant of GdL2 was calculated from the mL NaOH – pH data of the "out-of-cell" samples, which had equilibrium pH values in the range of 2.0 – 4.0. For the complete characterization of the Gd^{3+}-L2 system, the GdL2 complex (which is formed completely at about pH=3.5) was titrated with 0.2 M NaOH solution in the pH range 3.5 – 12.0.

During these titrations, base consumption was observed at about pH>3.5 and pH>8.0, which indicated the deprotonation of the Gd(HL) and GdL species, respectively. These processes correspond the dissociation of H^+ ion from the carboxylate and the alcoholic -OH group of the 2-hydroxypropanoic pendant arm (Eqs. (S3) and (S4)). A pH-potentiometric titration of GdL1 complex with 0.2 M NaOH solution was also performed to determine the deprotonation constant of the hydroxyl group in the methyl 2-hydroxypropanoate pendant arm (log$K_{Gd(L)H-1}$, Table S1).

By taking into account the protonation constants of the ligand L2, the stability constant of the *Gd(H_2L2) intermediate and the stability and protonation constant of the GdL2 complex, the species distribution of the Gd^{3+} - L2 system was calculated (Figure S1).

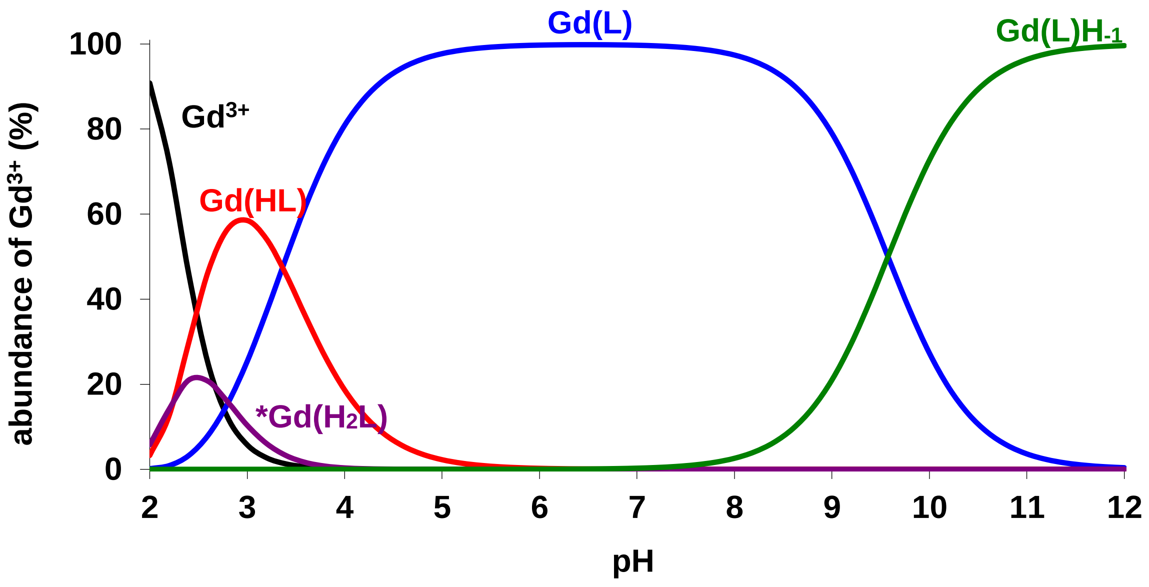

Figure S1. Species distribution diagram of the Gd^{3+}- L2 system ([Gd^{3+}]=[L2]=1.0 mM, 0,15 M NaCl, 298K)

Then, the stability and protonation constants of CuL2 were determined by spectrophotometry. The equilibrium reaction (Eq. (S6)) was studied in the [H^+] range of 0.01 – 1.0 M. The ionic strength of these samples was not constant at [H^+]>0.15 M ([H^+]≤0.15 M, I=[Na^+] + [H^+] = 0.15 M). In these sample the formation of Cu^{2+}, Cu(H_2L), Cu(H_3L) and H_xL species was assumed.

$$Cu^{2+} + H_xL \rightleftharpoons [Cu(H_{x-y}L)] + yH^+ \qquad (S6)$$

where x=5 and 6; y=3 and 4. Some characteristic absorption spectra are shown in Figure S2.

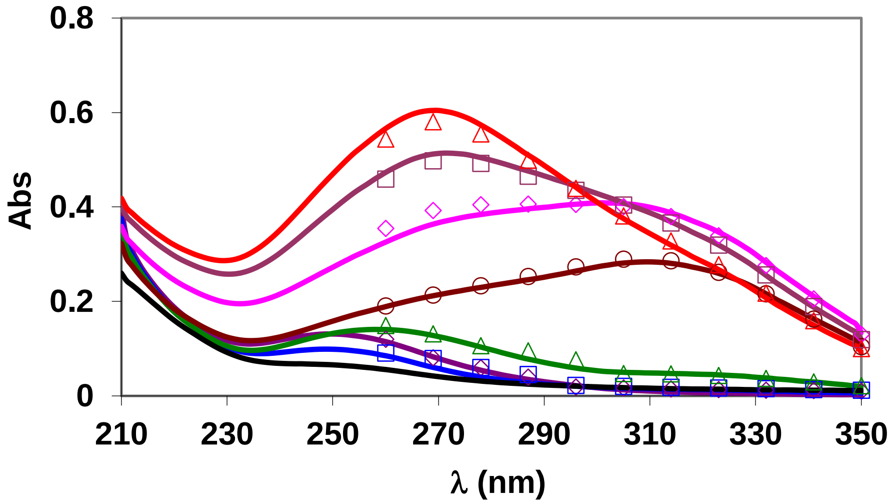

Figure S2. Absorbtion spectra of Cu^{2+}–L2 system. The solid lines and the open symbols represent the experimental and the calculated absorbance values, respectively. ($[Cu^{2+}]$=[L2]=0.72 mM, $[H^+]$ = **1.0** (□), **0,60** (◇), **0.31** (△), **0.10** (○), **0.043** (◇), **0.023** (□) and **0.011 M** (△), **$[Cu^{2+}]$=0.72 mM**, I=$[Na^+]$+$[H^+]$=0.15, $[H^+]$≤0.15 M, l=0.2 cm, 25°C).

The stability and protonation constants of the Ca^{II}-, Zn^{II}-, Cu^{II}- and Gd^{III}-L2 complexes are shown and compared with those of HPADO3A, HPDO3A, DOTA and BT-DO3A complexes in Table S1. The stability constants of the Ca^{II}, Zn^{II}, Cu^{II} and Gd^{III} complexes formed with L2, HPADO3A and BT-DO3A (Table S1) are very similar and about 1 – 5 orders of magnitude lower than those of the corresponding DOTA and HPDO3A complexes, which might be explained by the lower logK_1^H and logK_2^H values of L2, HPADO3A and BT-DO3A ligands due to the formation of the Na^I-complexes. Although the stability constants obtained for CuL2 and Cu(HPADO3A) are significantly higher than that of Cu(BT-DO3A) published earlier in literature,[5] it can be assumed that pH-potentiometric method used for the determination of the stability constant of Cu(BT-DO3A) cannot provide reliable information for the estimation of the logK_{CuL} value due to complete formation of $Cu(H_2L)$ species and the negligible amount of free Cu^{2+} ion at pH>1.7.

The equilibrium constants characterizing the protonation of the carboxylate group in the 2-hydroxypropanoic pendant arm of ZnL2 (ZnH_2L2), CuL2 (CuH_2L2) and GdL2 (GdHL2) complexes are very similar to that of the free L2 ligand (logK_4^H), which indicate that the extra carboxylate group does not coordinate the metal ions. Interestingly, the stability constant of the diprotonated *$Gd(H_2L2)$ intermediate (log$K_{Gd(H2L)}$) is similar to that of *$Gd(H_2DOTA)$ and about 1.5 – 2 log$K$ unit higher than that of *$Gd(H_2HPADO3A)$ and *$Gd(H_2HPDO3A)$, which might be interpreted by the coordination of the Gd^{III} ion by the four carboxylate groups of L2 resulting in the formation of *$Gd(H_2L1)$ intermediate similar to *$Gd(H_2DOTA)$.

Table S1. Protonation constants of L2, HPADO3A, HPDO3A, DOTA and BT-DO3A ligand, the stability and protonation constants of Ca^{II}-, Zn^{II}-, Cu^{II}- and Gd^{III}-complexes at 25°C

	**L2**	**HPADO3A**[a]	**HPDO3A**[b,c]	**DOTA**[f]	**BT-DO3A**[d]
**I**	0.15 M NaCl		0.1 M Me_4NCl	0.1 M NaCl	0.1 M NaCl
$\log K_1^H$	8.95 (3)	8.96	11.96	9.37	9.46
$\log K_2^H$	8.95 (2)	9.07	9.43	9.14	9.36
$\log K_3^H$	4.22 (3)	4.22	4.30	4.63	4.17
$\log K_4^H$	**3.74 (3)**	2.64	3.26	3.91	3.02
$\log K_5^H$	2.47 (4)	1.25	–	–	–
$\log K_5^H$	1.75 (4)	–	–	–	–
**CaL**	**11.63 (1)**	**12.13**	**14.83**	**16.37**[e]	**12.1**
CaHL	5.20 (5)	4.67	–	3.60[e]	–
$Ca(L)H_{-1}$	–	11.50	–	–	–
**ZnL**	**17.81 (6)**	**17.18**	**19.37**	**18.7**[e]	**17.0**
ZnHL	3.86 (5)	3.67	3.7	5.33[e]	4.3
ZnH_2L	**3.32 (3)**	2.87	–	3.96[e]	–
ZnH_3L	2.29 (5)	–	–	–	–
$Zn(L)H_{-1}$	–	10.79 (5)	–	10.62[e]	–
***CuL**	**21.87 (6)**	**21.53**	**22.84**	**22.72**[e]	**19.1**
CuHL	3.84 (2)	4.00	3.72	4.45[e]	3.8
CuH_2L	**3.36 (2)**	1.24	2.3	3.92[e]	2.4
CuH_3L	1.36 (8)	–	–	–	–
$Cu(L)H_{-1}$	–	10.55	–	–	–
**GdL**	**19.26 (3)**	**18.41**	**23.8**	**24.7**	**18.7**
**GdHL**	**3.36 (3)**	–	–	–	–
****GdH_2L**	6.73 (3)	5.72	5.10	6.07	4.53
**$Gd(L)H_{-1}$**	**9.58 (3)**	**6.73**	**11.36**[d]	–	**9.48**

[a] Ref. [11]; 0.1 M KCl, 25°C; [b]Ref. [13], 0.1 M Me_4NCl, 25°C; [c] Ref. [14]; [d] Ref. [5], 0.1 M NaCl, 25 °C; [e] Ref. [15], 0.1 M KCl, 25°C; [f] Ref. [16] 0.1 M NaCl, 25°C; GdL1: $\log K_{Gd(L)H-1}$=9.36 (6); *obtained by spectrophotometry; **stability constants of the protonated *$Gd(H_2L)$ out-of-cage complex (intermediate): $K_{Gd(H2L)}=[Gd(H_2L)]/[Gd^{3+}][H_2L]$, 0.15 M NaCl, 25°C

The protonation constant of the alkoxide-O^- donor atom ($\log K_{Gd(L)H-1}$) in GdL1 and GdL2 complexes are about 2 orders of magnitude lower than that of Gd(HPDO3A) due to the electron withdrawing effect of the carboxylate and ester pendants of the 2-hydroxypropanoic/ate side chain in GdL1 and GdL2. On the other hand, these values are about 3 orders of magnitude higher than that of

Gd(HPADO3A) ($\log K_{Ga(L)H-1}$=6.73),[11] due to the significantly higher electron withdrawing effect of the amide functional group in the 2-hydroxypropanamide side chain of Gd(HPADO3A).

2. Kinetic inertness of GdL2

In order to investigate the kinetic inertness of GdL2, the dissociation reactions of GdL2 were followed by ^{1}H-NMR relaxometry in the presence of large acid excess ([HCl]= 0.01 – 1.0 M) to guarantee the pseudo-first-order kinetic conditions. The R_1^{obs} values as a function of time for the dissociation reactions of GdL1 are shown in Figure S3.

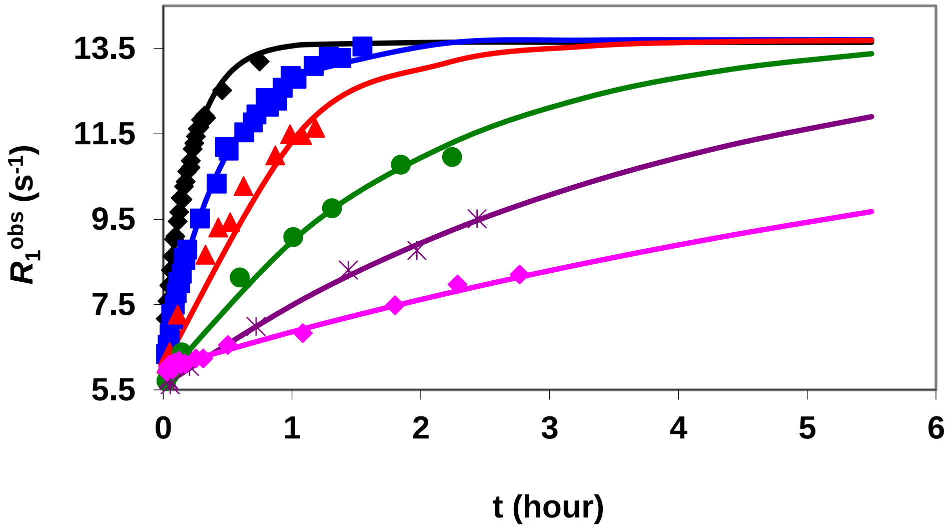

Figure S3. r_{1p} values of GdL2 as a function of time in the presence of **0.70 (◆)**, **0.29 (■)**, **0.16 (▲)**, **0.06 (●)**, **0.025 (✳)** and **0.010** (◆) M HCl, ([GdL2]=1.0 mM, [H$^+$]≤0.15 M→[Na$^+$]+[H$^+$]=0.15 M, 25°C)

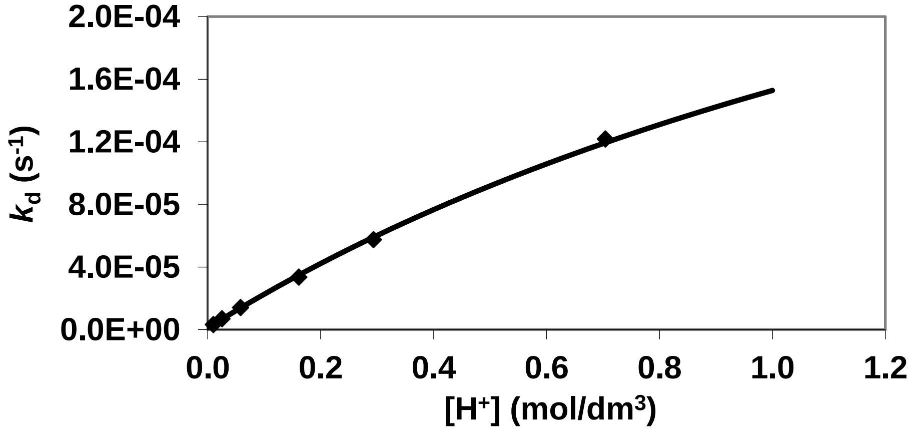

Figure S4. Pseudo-first-order rate constant (k_d) characterizing the dissociation of GdL2 as a function of [H$^+$] ([GdL2]=1.0 mM, [H$^+$]≤0.15 M→[Na$^+$]+[H$^+$]=0.15 M, 25°C).

In the presence of HCl excess, the dissociation of GdL2 can be treated as a pseudo-first-order process and the rate of the reaction can be expressed by Eq. (S7)

$$-\frac{d[GdL]_t}{dt} = k_d[GdL]_{tot} \qquad (S7)$$

where k_d is a pseudo-first-order rate constant, $[GdL]_t$ and $[GdL]_{tot}$ are the concentrations of the GdL species at time *t* and the total concentration of the complex, respectively. The rates of the dissociation reactions were determined at different concentrations of HCl ([HCl]=0.01 – 1.0 M). The k_d values as a function of $[H^+]$ are shown in Figure S4.

As it is shown in Figure S4, k_d pseudo-first order rate constants increase with the concentration of H^+, which can be explained by the proton assisted dissociation of GdL2. The proton assisted dissociation of GdL2 might occur by the equilibrium formation of a protonated *Gd(H_2L2) intermediate (Eq. (S8)), which dissociates spontaneously (Eq. (S9)).

$$Gd(HL2) + H^+ \overset{K^H_{Gd(HL)}}{\rightleftharpoons} Gd(H_2L2) \qquad (S8)$$

$$K^H_{Gd(HL)} = \frac{[Gd(H_2L)]}{[Gd(HL)][H^+]}$$

$$^*Gd(H_2L2) \xrightarrow{k_{GdH2L}} Gd^{3+} + H_xL2 \qquad (S9)$$

k_{GdH2L} is the rate constant characterizing the dissociation of *Gd(H_2L2) intermediate. The K^H_{GdHL} is the protonation constant of the protonated Gd(HL2) species, which predominates at pH<3.0 (Figure S1). By considering the dissociation of the *Gd(H_2L2) intermediate (Eq. (S8)) and the rate of dissociation of GdL2 (Eq. (7)), the pseudo-first-order rate constant (k_d) can be expressed by Eq. (10).

$$-\frac{d[GdL]}{dt} = k_{GdH_2L}[Gd(H_2L)] \qquad (S10)$$

By taking into account the total concentration of the complex ($[GdL2]_{tot}$=[Gd(HL2)]+[*Gd(H_2L2)]), the protonation constants of Gd(HL2) (K^H_{GdHL}, Eq. (8)) and Eq. (10), the pseudo-first-order rate constant (k_d) can be expressed as follows:

$$k_d = \frac{k_1[H^+]}{1+K^H_{GdHL}[H^+]} \qquad (S11)$$

where $k_1 = k_{GdH2L} \times K^H_{GdHL}$ are the rate constants characterizing the proton assisted dissociation of Gd(HL2). k_1 value of Gd(HL2) were calculated by fitting of the kinetic data (Figure S12) to Eq. (S11).

3. X-ray diffraction studies of $[Gd(L2)H_{-1}(OH^{-})]^{3-}$ complex

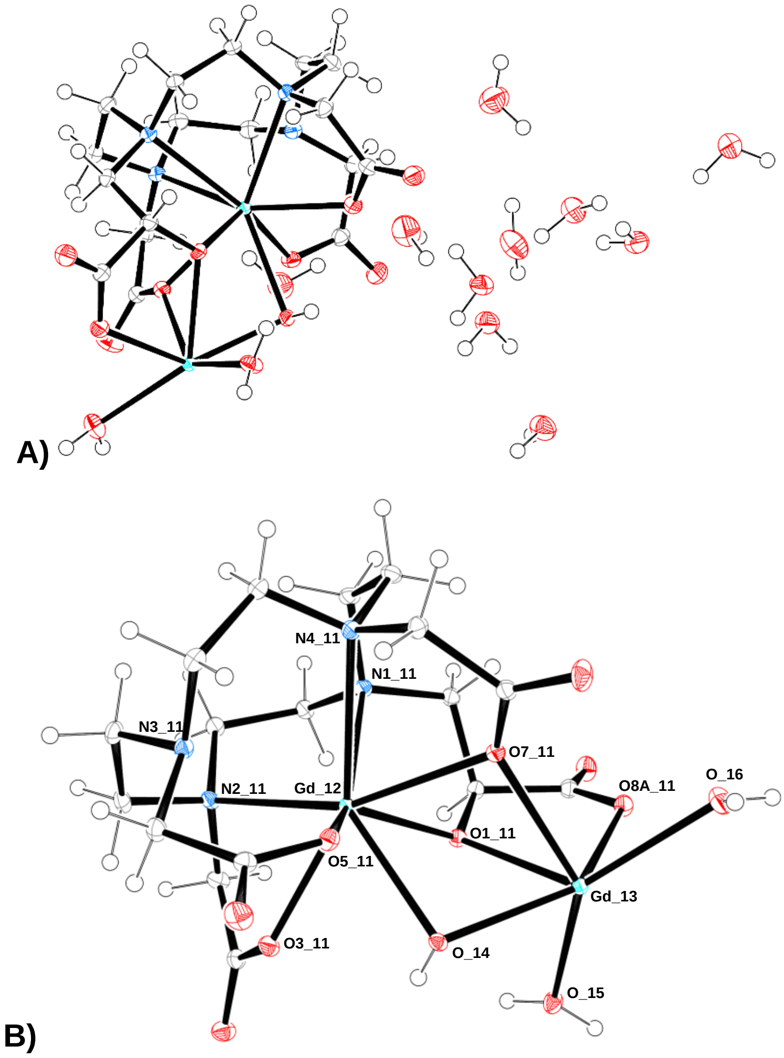

Figure S5. Ellipsoids representation of crystallographic asymmetric unit content (ASU) for dimeric $[Gd(L2)H_{-1}(OH^{-})]^{3-}$ (50% probability): **A)** complete ASU content; **B)** solvent water molecules omitted for clarity. Naming scheme used for Gd^{III}-ion coordination spheres is reported.

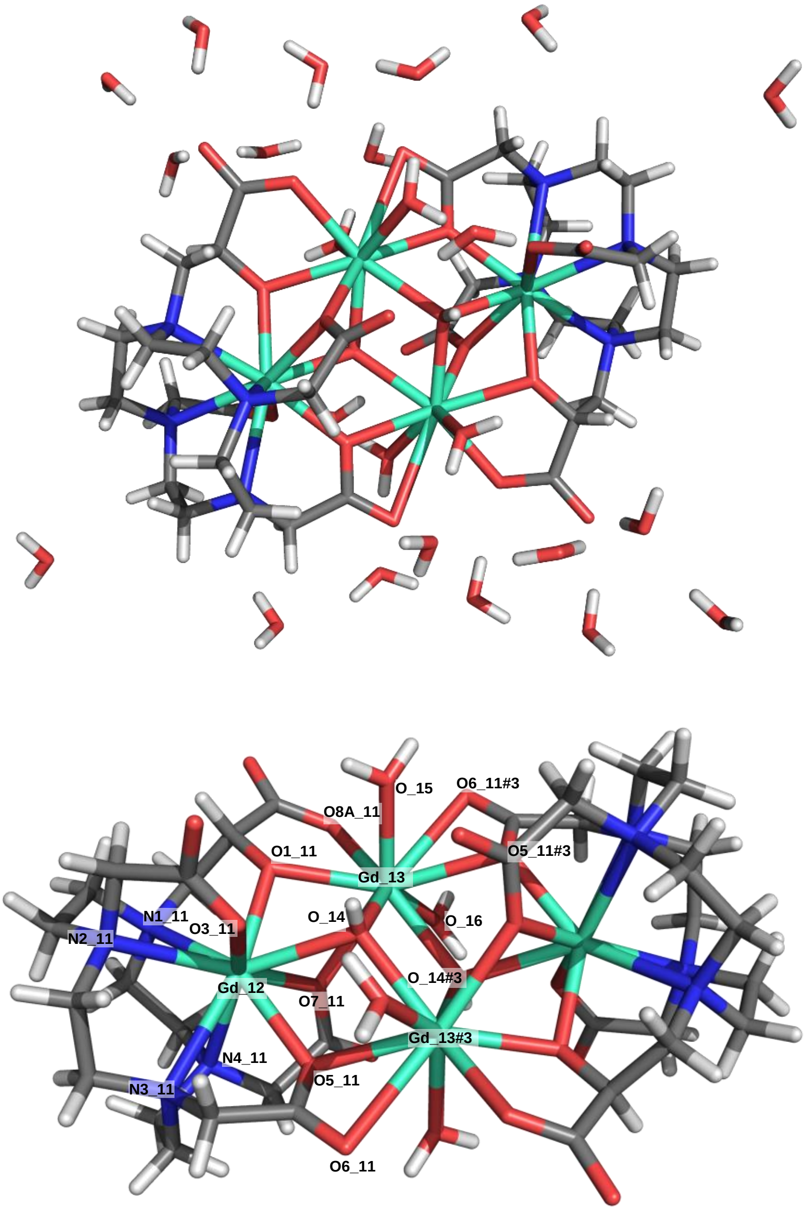

Figure S6. Sticks representation of dimeric $[Gd(L2)H_{-1}(OH^{-})]^{3-}$. Hydrogen bonded water molecules surrounds the capsule (omitted for clarity). Naming scheme used for Gadolinium coordination spheres is reported. A crystallographic inversion center lies on dimeric $[Gd(L2)H_{-1}(OH^{-})]^{3-}$ barycenter.

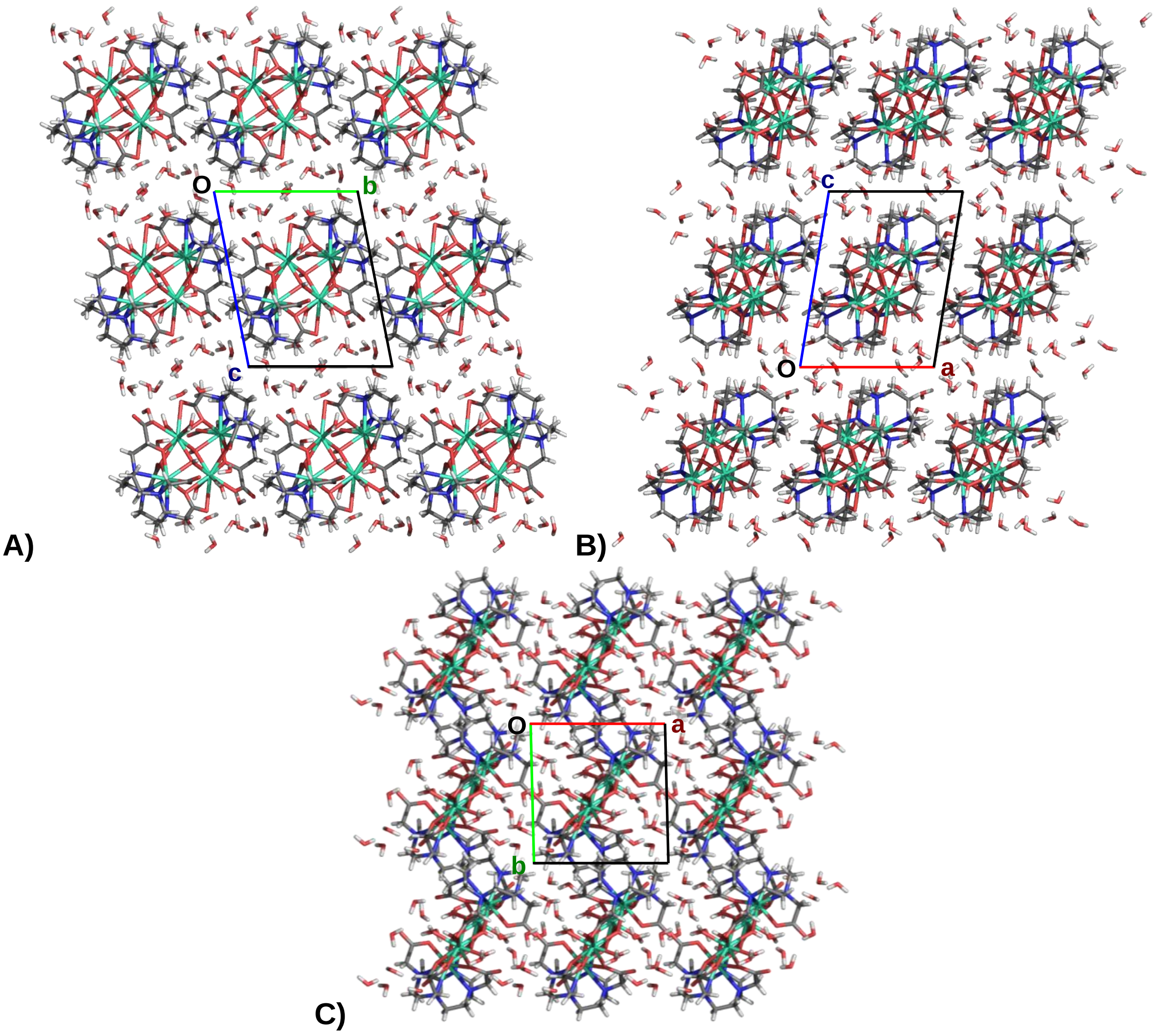

Figure S7. Crystal packing of dimeric $[Gd(L2)H_{-1}(OH^{-})]^{3-}$ along crystallographic *a*, *b* and *c* axis.

Table S2. Crystallographic data and refinement details for dimeric $[Gd(L2)H_{-1}(OH^{-})]^{3-}$.

	$[Gd_4(C_{17}H_{25}N_4O_9)_2(OH)_2(H_2O)_4 \cdot 20H_2O]$
CCDC Number	2087813
Chemical Formula	$C_{34}H_{100}Gd_4N_8O_{44}$
Formula weight (g/mol)	1954.21
Temperature (K)	100(2)
Wavelength (Å)	0.700
Crystal system	Triclinic
Space Group	*P*-1
Unit cell dimensions	a = 10.541(2) Å
	b = 11.172(2) Å
	c = 14.209(3) Å
	α = 78.78(3)°
	β = 80.23(3)°
	γ = 86.89(3)°
Volume (Å^3)	1617.1(6)
Z	1
Density (calculated) (g·cm^{-3})	2.007
Absorption coefficient (mm^{-1})	3.979
F(000)	968
Crystal size (mm^3)	0.12 x 0.08 x 0.05
Crystal habit	Colorless rods
Theta range for data collection	2.11° to 30.00°
Resolution (Å)	0.70
Index ranges	$-14 \le h \le 13$
	$-15 \le k \le 15$
	$-20 \le l \le 20$
Reflections collected	49466
Independent reflections (data with I>2σ(I))	9571 (9542)
Data multiplicity (max resltn)	5.02 (4.38)
I/σ(I) (max resltn)	27.64 (25.43)
R_{merge} (max resltn)	0.0485 (0.0502)
Data completeness (max resltn)	97.2% (93.6%)
Refinement method	Full-matrix least-squares on F^2
Data / restraints / parameters	9571 / 37 / 485
Goodness-of-fit on F^2	1.063
Δ/σ_{max}	0.002
Final R indices [I>2σ(I)]^a	R_1 = 0.0207, wR_2 = 0.0540
R indices (all data)^a	R_1 = 0.0207, wR_2 = 0.0542
Largest diff. peak and hole (e·Å^{-3})	0.898 and -1.654
R.M.S.D. from mean (e·Å^{-3})	0.126

^{a} $R_1 = \Sigma \left||Fo|-|Fc|\right| / \Sigma |Fo|$, $wR_2 = \{\Sigma [w(Fo^2 - Fc^2)^2] / \Sigma [w(Fo^2)^2]\}^{1/2}$

Table S3. Selected bond distances and angles (Å and degrees) for coordination spheres of the crystallographically independent Gd^{III} centers in dimeric $[Gd(L2)H_{-1}(OH^{-})]^{3-}$. (Gd_12 and Gd_13). Naming scheme is reported in Figure S6.

**Gd_12 - diGdHPDO4A**							
**Distances**	**(Å)**	**Angles**	**(°)**	**Angles**	**(°)**	**Angles**	**(°)**
Gd_12-O1_11	2.289(2)	O3_11-Gd_12-O1_11	94.35(6)	N1_11-Gd_12-O7_11	88.11(5)	O5_11-Gd_12-O3_11	86.61(6)
Gd_12-O3_11	2.406(2)	O5_11-Gd_12-O1_11	130.73(5)	N2_11-Gd_12-O7_11	155.19(5)	O7_11-Gd_12-O3_11	138.50(5)
Gd_12-O5_11	2.357(2)	O7_11-Gd_12-O1_11	69.69(5)	N3_11-Gd_12-O7_11	117.83(5)	N1_11-Gd_12-O3_11	121.98(5)
Gd_12-O7_11	2.444(2)	N1_11-Gd_12-O1_11	67.72(6)	N4_11-Gd_12-O7_11	63.29(5)	N2_11-Gd_12-O3_11	63.55(5)
Gd_12-N1_11	2.659(2)	N2_11-Gd_12-O1_11	102.20(6)	O_14-Gd_12-O7_11	70.69(5)	N3_11-Gd_12-O3_11	85.83(6)
Gd_12-N2_11	2.662(2)	N3_11-Gd_12-O1_11	167.47(5)	Gd_13-Gd_12-O7_11	43.04(4)	N4_11-Gd_12-O3_11	150.67(5)
Gd_12-N3_11	2.749(2)	N4_11-Gd_12-O1_11	114.22(6)	Gd_13#1-Gd_12-O7_11	68.76(4)	O_14-Gd_12-O3_11	67.81(5)
Gd_12-N4_11	2.703(2)	O_14-Gd_12-O1_11	67.88(5)	N3_11-Gd_12-N2_11	66.67(6)	Gd_13-Gd_12-O3_11	100.28(4)
Gd_12-O_14	2.461(1)	Gd_13-Gd_12-O1_11	40.37(4)	N4_11-Gd_12-N2_11	102.02(5)	Gd_13#1-Gd_12-O3_11	76.71(4)
Gd_12-Gd_13	3.621(1)	Gd_13#1-Gd_12-O1_11	98.75(4)	O_14-Gd_12-N2_11	129.36(5)	O7_11-Gd_12-O5_11	77.43(5)
Gd_12-Gd_13#1	4.060(1)	N2_11-Gd_12-N1_11	67.33(5)	Gd_13-Gd_12-N2_11	140.49(4)	N1_11-Gd_12-O5_11	147.25(5)
		N3_11-Gd_12-N1_11	101.59(6)	Gd_13#1-Gd_12-N2_11	135.99(4)	N2_11-Gd_12-O5_11	121.52(5)
		N4_11-Gd_12-N1_11	67.46(5)	O_14-Gd_12-N3_11	123.28(5)	N3_11-Gd_12-O5_11	61.80(5)
		O_14-Gd_12-N1_11	135.10(5)	O_14-Gd_12-N4_11	127.84(5)	N4_11-Gd_12-O5_11	79.80(5)
		Gd_13-Gd_12-N1_11	98.33(4)	Gd_13-Gd_12-N4_11	105.77(4)	O_14-Gd_12-O5_11	67.13(5)
		Gd_13#1-Gd_12-N1_11	156.44(4)	Gd_13#1-Gd_12-N4_11	103.90(4)	Gd_13-Gd_12-O5_11	90.92(4)
		N4_11-Gd_12-N3_11	64.83(5)	Gd_13-Gd_12-O_14	39.91(4)	Gd_13#1-Gd_12-O5_11	33.73(4)
		Gd_13-Gd_12-N3_11	151.83(4)	Gd_13#1-Gd_12-O_14	33.51(4)		
		Gd_13#1-Gd_12-N3_11	93.49(4)			Gd_13#1-Gd_12-Gd_13	61.90(1)
Symmetry transformations used to generate equivalent atoms: #1 -x+1,-y+1,-z+1							

**Gd_13 - diGdHPDO4A**							
**Distances**	**(Å)**	**Angles**	**(°)**	**Angles**	**(°)**	**Angles**	**(°)**
Gd_13-O1_11	2.392(2)	O5_11#3-Gd_13-O1_11	146.82(5)	O_14-Gd_13-O8A_11	135.77(5)	O7_11-Gd_13-O6_11#3	144.70(5)
Gd_13-O5_11#3	2.475(2)	O6_11#3-Gd_13-O1_11	124.75(5)	O_15-Gd_13-O8A_11	85.25(6)	O8A_11-Gd_13-O6_11#3	66.89(5)
Gd_13-O6_11#3	2.828(2)	O7_11-Gd_13-O1_11	67.47(5)	O_16-Gd_13-O8A_11	76.35(5)	O_14-Gd_13-O6_11#3	142.17(5)
Gd_13-O7_11	2.480(2)	O8A_11-Gd_13-O1_11	67.74(5)	O_14#3-Gd_13-O8A_11	151.13(5)	O_15-Gd_13-O6_11#3	71.27(5)
Gd_13-O8A_11	2.409(2)	O_14-Gd_13-O1_11	68.19(5)	Gd_12-Gd_13-O8A_11	99.92(4)	O_16-Gd_13-O6_11#3	63.96(5)
Gd_13-O_14	2.344(2)	O_15-Gd_13-O1_11	75.22(5)	Gd_13#1-Gd_13-O8A_11	163.40(4)	O_14#3-Gd_13-O6_11#3	106.13(5)
Gd_13-O_15	2.386(2)	O_16-Gd_13-O1_11	130.47(5)	O_15-Gd_13-O_14	80.25(6)	Gd_12-Gd_13-O6_11#3	162.99(3)
Gd_13-O_16	2.470(2)	O_14#3-Gd_13-O1_11	128.65(5)	O_16-Gd_13-O_14	139.32(6)	Gd_13#1-Gd_13-O6_11#3	129.52(3)
Gd_13-O_14#3	2.425(2)	Gd_12-Gd_13-O1_11	38.29(4)	O_14#3-Gd_13-O_14	67.38(6)	O8A_11-Gd_13-O7_11	94.45(5)
Gd_13-Gd_12	3.621(1)	Gd_13#1-Gd_13-O1_11	99.33(4)	Gd_12-Gd_13-O_14	42.33(3)	O_14-Gd_13-O7_11	71.99(5)
Gd_13-Gd_13#1	3.968(1)	O6_11#3-Gd_13-O5_11#3	48.39(5)	Gd_13#1-Gd_13-O_14	34.33(4)	O_15-Gd_13-O7_11	139.54(5)
		O7_11-Gd_13-O5_11#3	140.31(5)	O_16-Gd_13-O_15	135.20(5)	O_16-Gd_13-O7_11	83.07(6)
		O8A_11-Gd_13-O5_11#3	115.14(5)	O_14#3-Gd_13-O_15	120.11(5)	O_14#3-Gd_13-O7_11	75.39(5)
		O_14-Gd_13-O5_11#3	99.76(5)	Gd_12-Gd_13-O_15	97.82(4)	Gd_12-Gd_13-O7_11	42.26(3)
		O_15-Gd_13-O5_11#3	72.24(5)	Gd_13#1-Gd_13-O_15	101.92(5)	Gd_13#1-Gd_13-O7_11	70.32(3)
		O_16-Gd_13-O5_11#3	79.27(5)	O_14#3-Gd_13-O_16	75.63(5)	Gd_12-Gd_13-O_14#3	90.58(4)
		O_14#3-Gd_13-O5_11#3	65.92(5)	Gd_12-Gd_13-O_16	125.19(5)	Gd_13#1-Gd_13-O_14#3	33.05(4)
		Gd_12-Gd_13-O5_11#3	142.01(4)	Gd_13#1-Gd_13-O_16	107.24(5)		
		Gd_13#1-Gd_13-O5_11#3	81.42(4)			Gd_12-Gd_13-Gd_13#1	64.49(1)
Symmetry transformations used to generate equivalent atoms: #1 -x+1,-y+1,-z+1							

Table S4. Geometrical parameters of polar contacts found in dimeric $[Gd(L2)H_{-1}(OH^{-})]^{3-}$ crystal packing. Naming scheme is reported in Figure S8.

diGdHPDO4A				
**D-H···A**	**d(D-H) (Å)**	**d(H···A) (Å)**	**d(D···A) (Å)**	**<(DHA) (°)**
O_14-H1_14...O3_11	0.73(3)	2.35(3)	2.715(2)	113(3)
O_15-H1_15...O_24#1	0.869(5)	1.912(7)	2.778(2)	174(3)
O_15-H2_15...O4_11#2	0.869(5)	1.875(9)	2.729(2)	167(3)
O_16-H1_16...O26#1	0.871(5)	2.009(7)	2.877(3)	174(4)
O_16-H2_16...O3_11#1	0.869(5)	2.29(4)	3.008(2)	141(5)
O_16-H2_16...O_25#1	0.869(5)	2.52(4)	3.137(3)	129(5)
C11_11-H11A_11...O_25#2	0.99	2.59	3.553(3)	164.1
C10_11-H10A_11...O_29#3	0.99	2.54	3.521(3)	171.4
C13_11-H13A_11...O_23	0.99	2.50	3.435(3)	156.9
C14_11-H14A_11...O_29#3	0.99	2.53	3.509(3)	170.5
C18_11-H18A_11...O_29#3	0.99	2.62	3.597(3)	169.4
C21_11-H21B_11...O8A_11#4	0.99	2.63	3.531(3)	150.7
C22_11-H22A_11...O_29#3	0.99	2.62	3.602(3)	172.0
C2_11-H2_11...O_25#2	1.00	2.40	3.377(3)	164.6
O_21-H1_21...O4A_11#2	0.87	1.893(6)	2.762(3)	177(3)
O_21-H2_21...O_25	0.87	1.859(9)	2.708(3)	165(3)
O_22-H1_22...O8A_11#1	0.87	1.949(6)	2.822(2)	179(3)
O_22-H2_22...O_28	0.87	2.035(12)	2.863(3)	158(3)
O_23-H1_23...O_24#5	0.87	1.941(17)	2.773(3)	158(4)
O_23-H2_23...O_21	0.87	2.020(11)	2.869(3)	163(3)
O_24-H1_24...O_30#6	0.87	1.945(16)	2.769(3)	158(4)
O_24-H2_24...O_27	0.87	1.962(9)	2.821(3)	169(4)
O_25-H1_25...O4_11	0.87	1.807(6)	2.676(2)	172(2)
O_25-H2_25...O8_11#1	0.87	1.865(13)	2.709(2)	162(4)
O_26-H1_26...O_27#6	0.87	2.049(19)	2.809(3)	145(3)
O_26-H2_26...O_22	0.87	1.971(18)	2.785(3)	155(4)
O_27-H1_27...O_23#6	0.87	1.843(7)	2.705(3)	170(3)
O_27-H2_27...O_6_11	0.87	2.43(3)	2.917(3)	116(3)
O_27-H2_27...O_22	0.87	1.988(11)	2.839(3)	165(3)
O_28-H1_28...O4A_11#1	0.87	1.877(11)	2.730(2)	166(4)
O_28-H2_28...O_30#7	0.87	1.986(8)	2.843(3)	168(3)
O_29-H1_29...O_26	0.87	1.899(14)	2.718(3)	156(3)
O_29-H2_29...O_21	0.87	1.875(10)	2.726(3)	165(3)
O_30-H1_30...O_28#8	0.87	1.931(11)	2.783(3)	166(4)
O_30-H2_30...O_29	0.87	1.887(7)	2.752(3)	172(4)
Symmetry transformations used to generate equivalent atoms: #1 -x+1,-y+1,-z+1; #2 -x+2,-y+1,-z+1; #3 x,y-1,z; #4 -x+1,-y,-z+1; #5 x+1,y,z; #6 -x+1,-y+1,-z+2; #7 x-1,y,z; #8 -x+1,-y+2,-z+2				

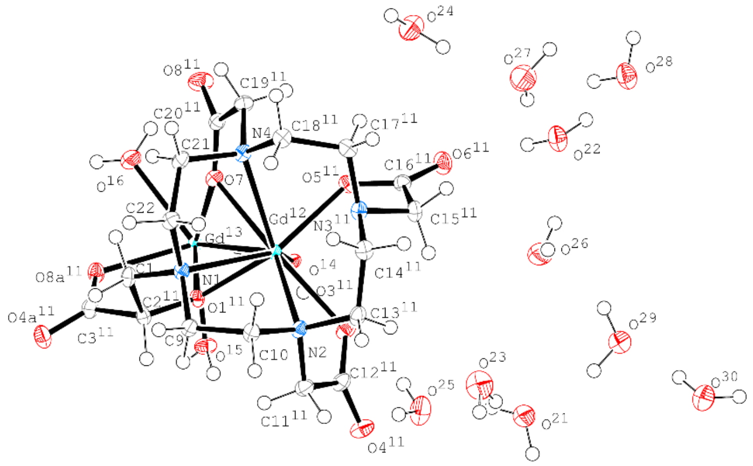

Figure S8. Naming scheme for the geometrical parameters of polar contacts found in dimeric $[Gd(L2)H_{-1}(OH^{-})]^{3-}$ crystal packing.

4. ^{1}H and ^{13}C NMR spectra of protected and deprotected ligands

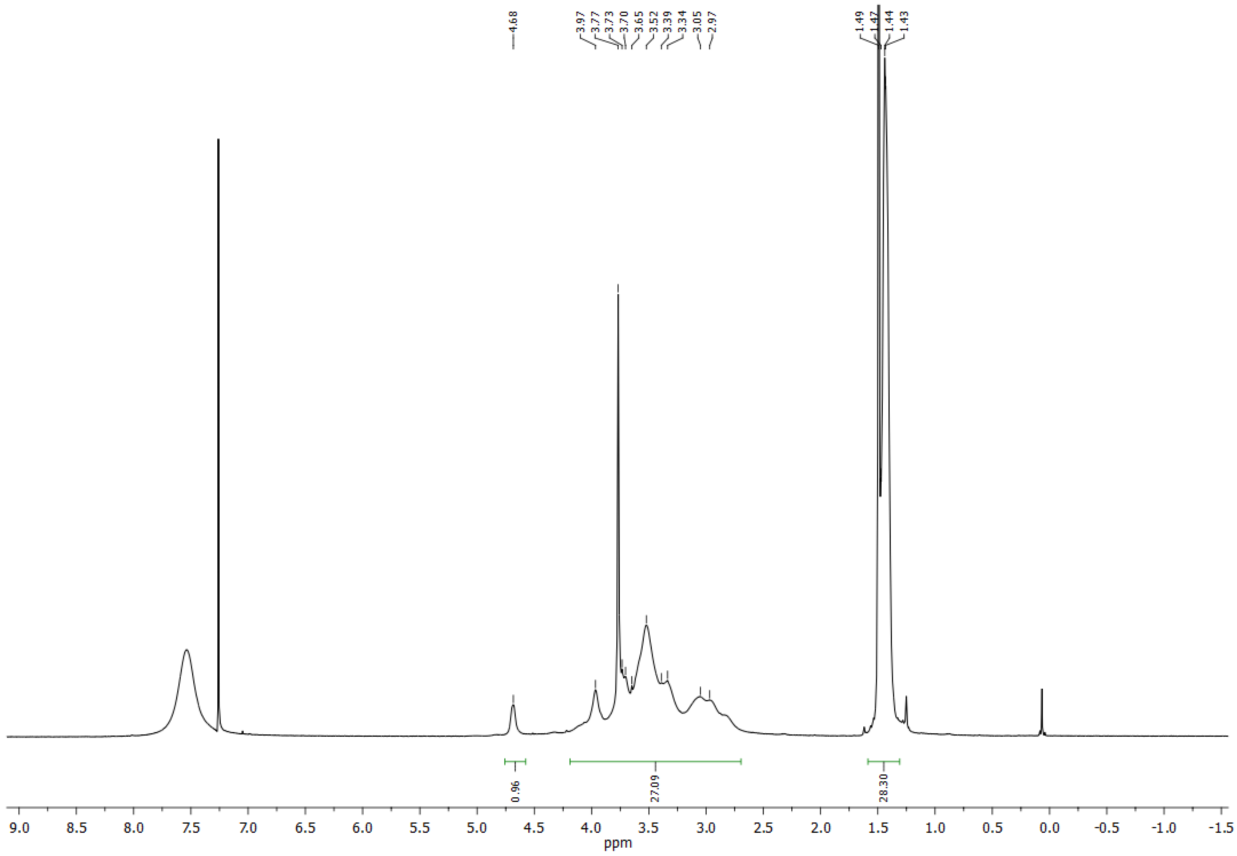

Fig. S9a ^{1}H NMR spectrum of **L1(O*t*Bu)$_3$**

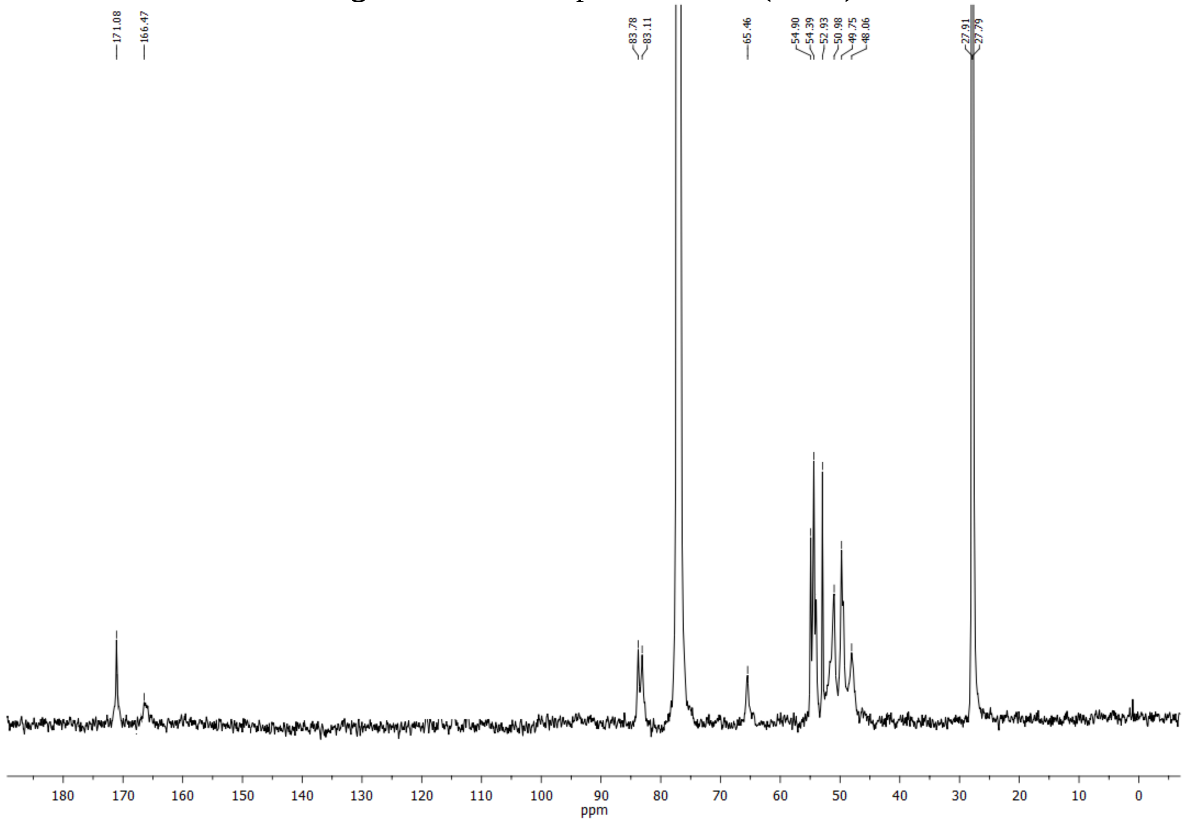

Fig. S9b ^{13}C NMR spectrum of **L1(O*t*Bu)$_3$**

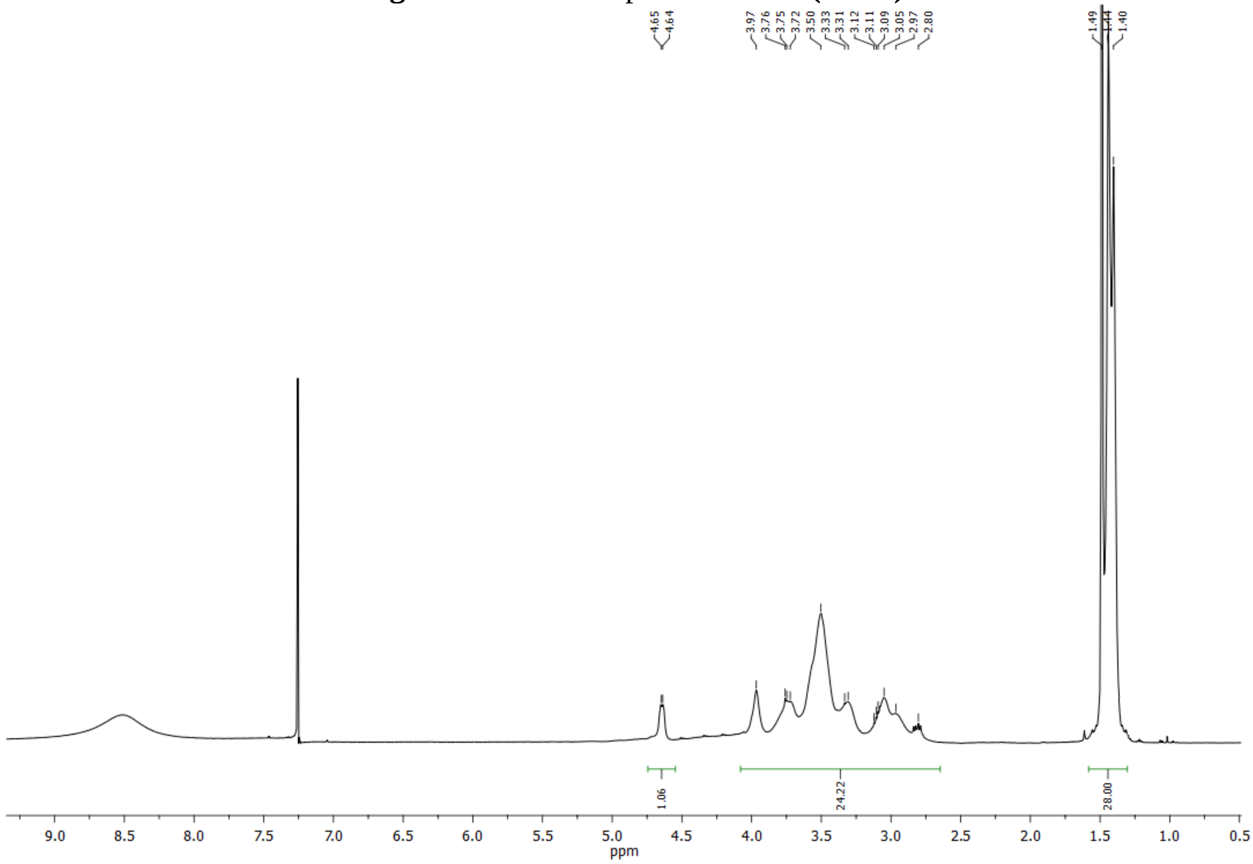

Fig. S10a ^{1}H NMR spectrum of **L2(O*t*Bu)$_3$**

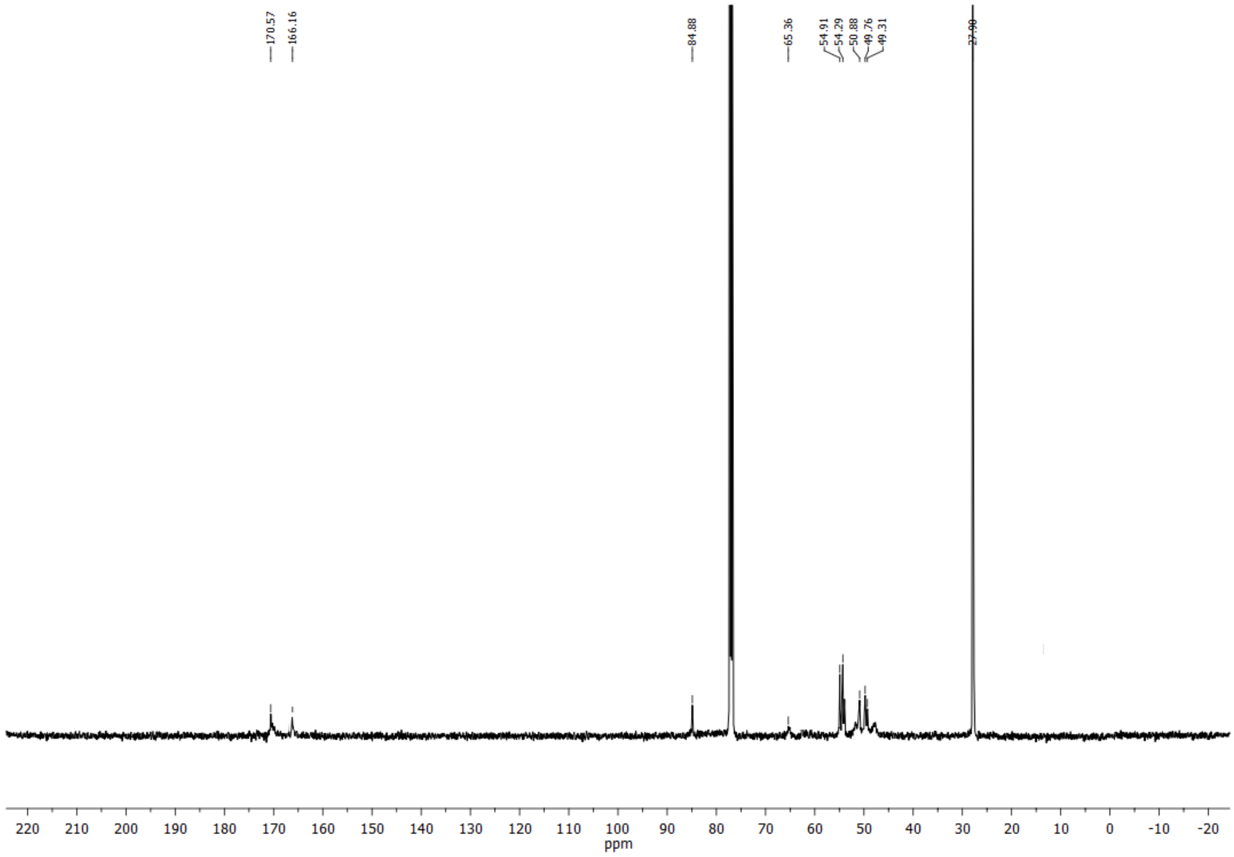

Fig. S10b ^{13}C NMR spectrum of **L2(O*t*Bu)$_3$**

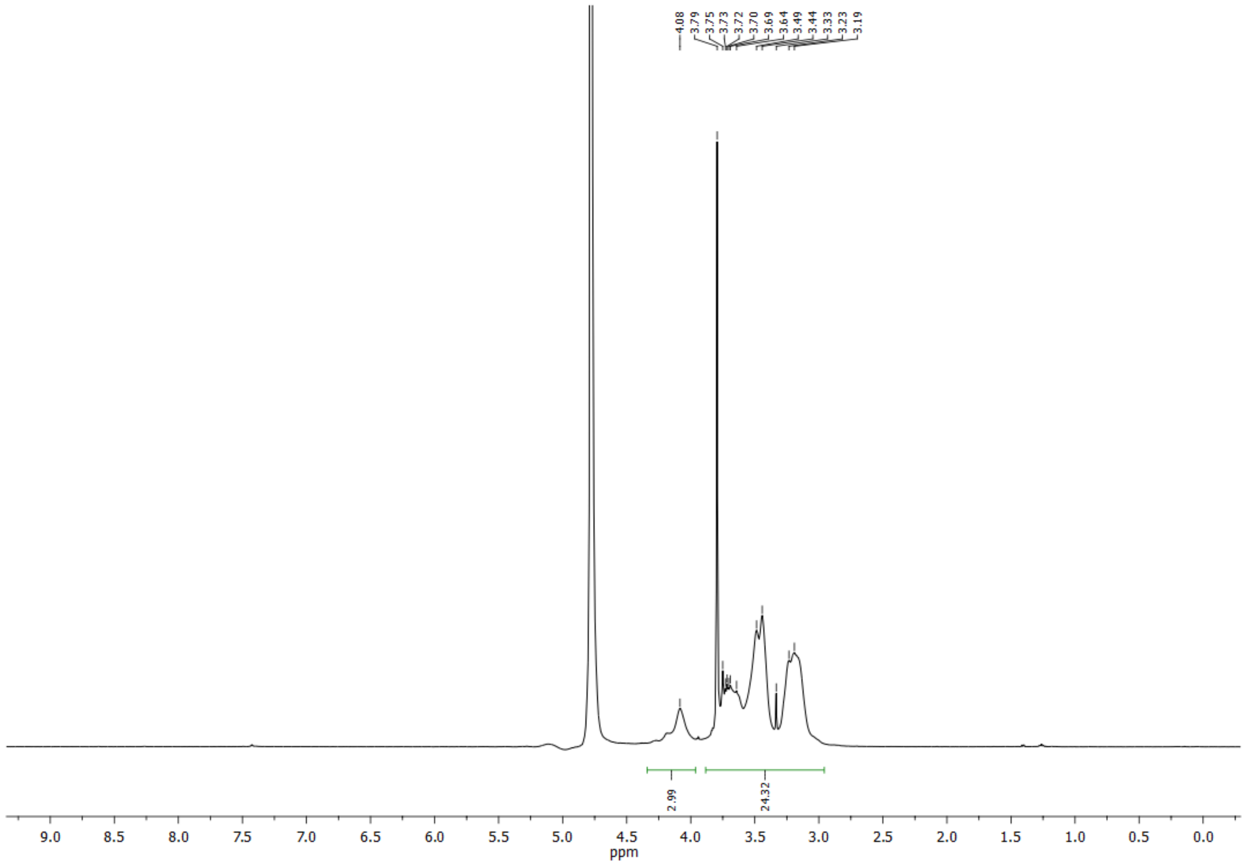

Fig. S11a ^{1}H NMR spectrum of **L1**

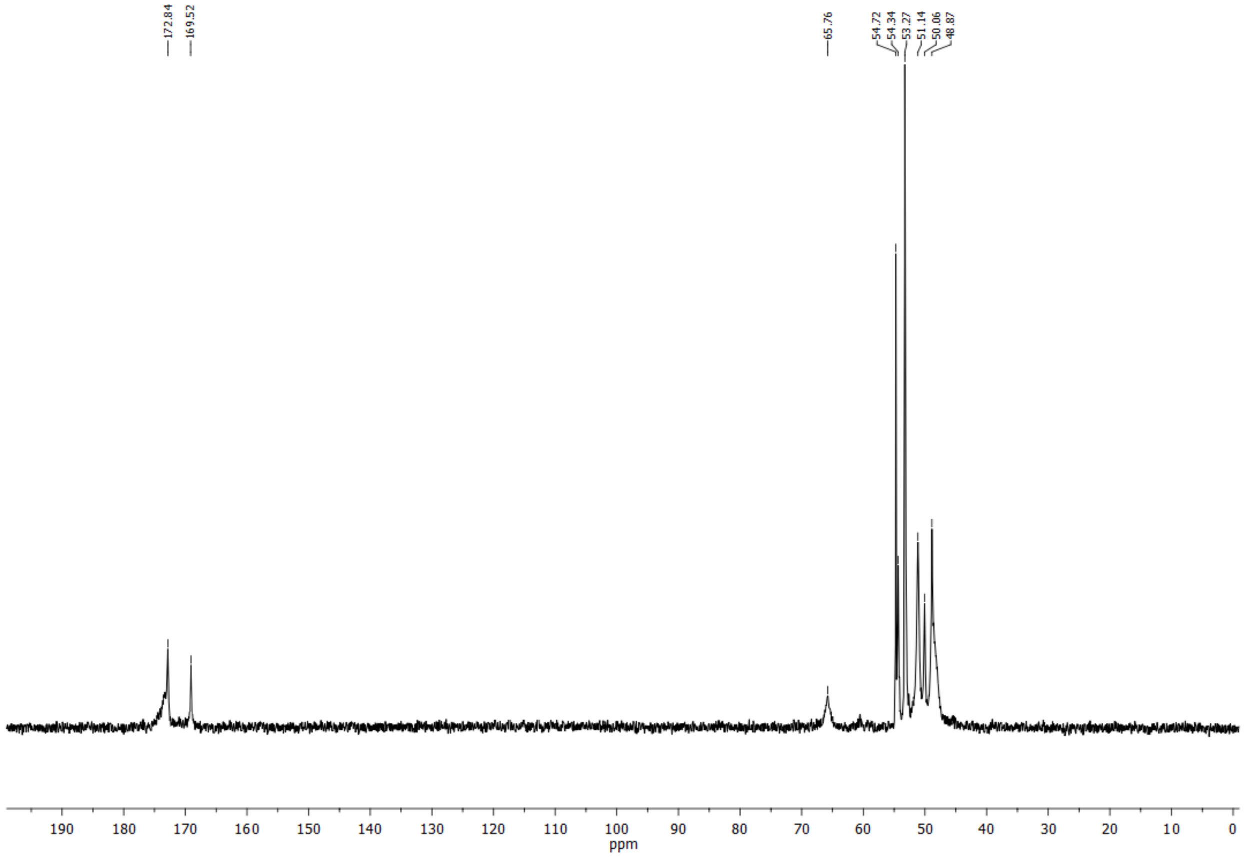

Fig. S11b ^{13}C NMR spectrum of **L1**

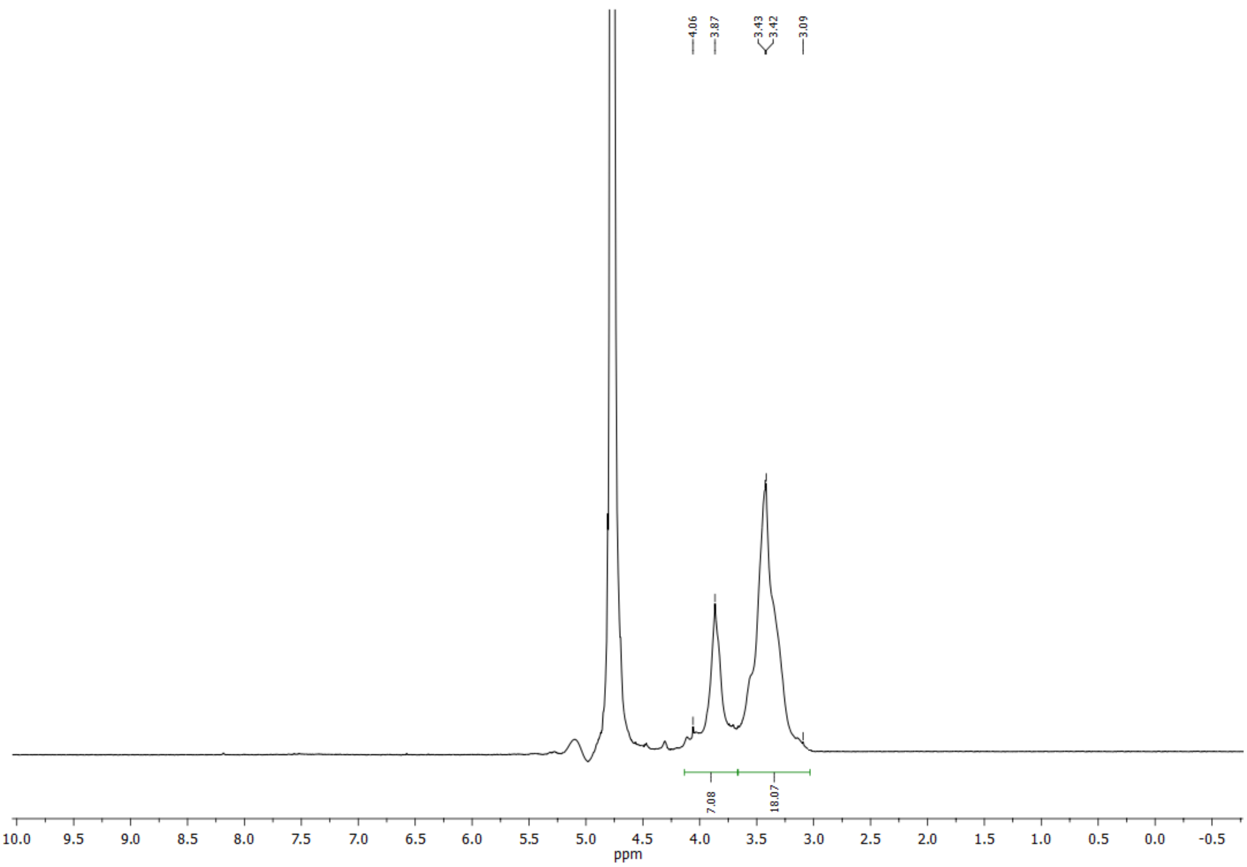

Fig. S12a ^{1}H NMR spectrum of **L2**

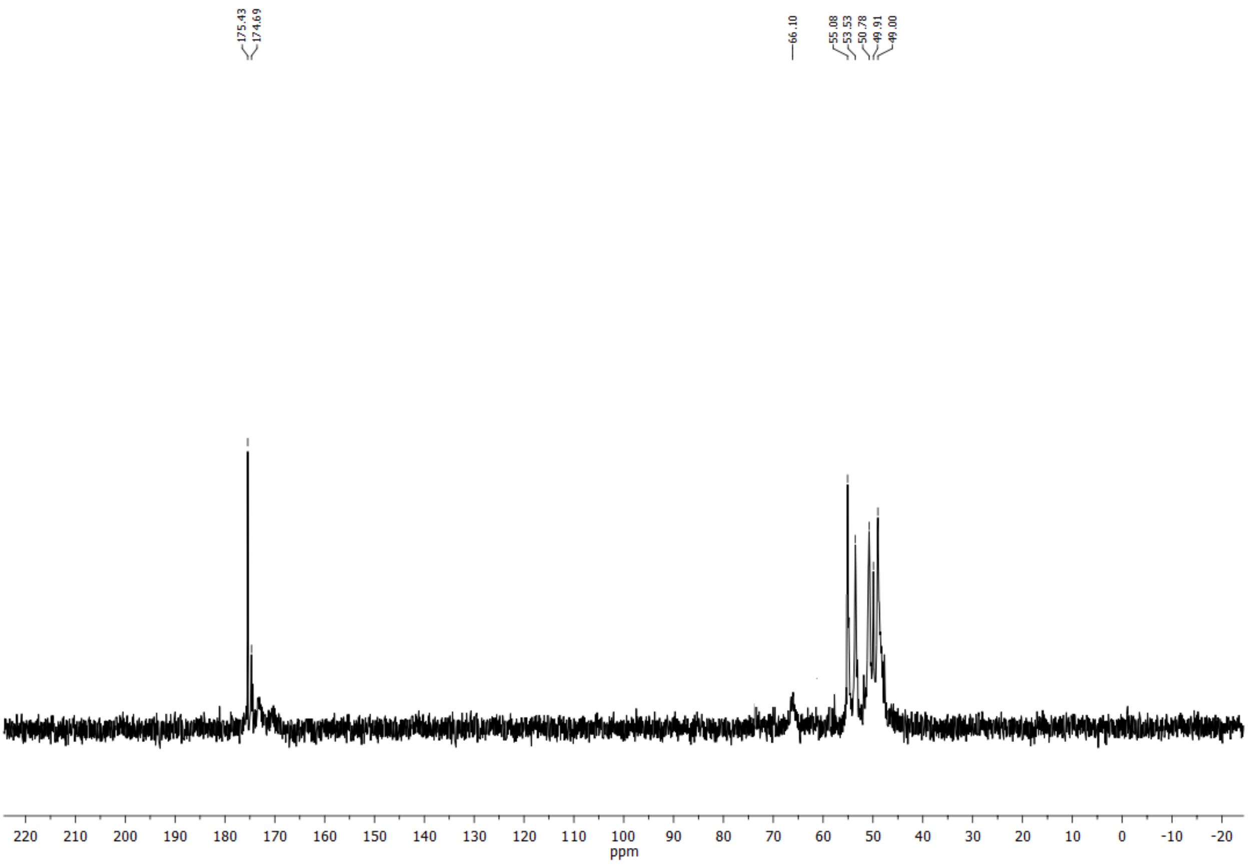

Fig. S12b ^{13}C NMR spectrum of **L2**

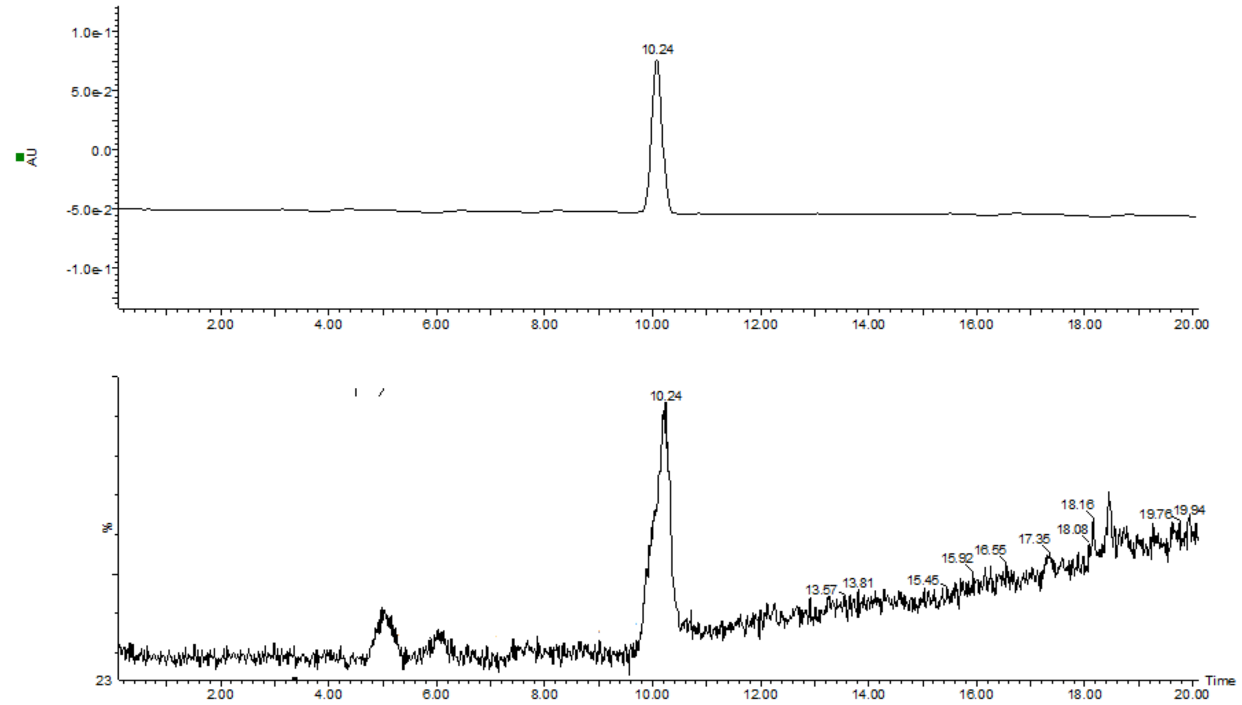

Fig. S13a HPLC-MS chromatogram of **L1**

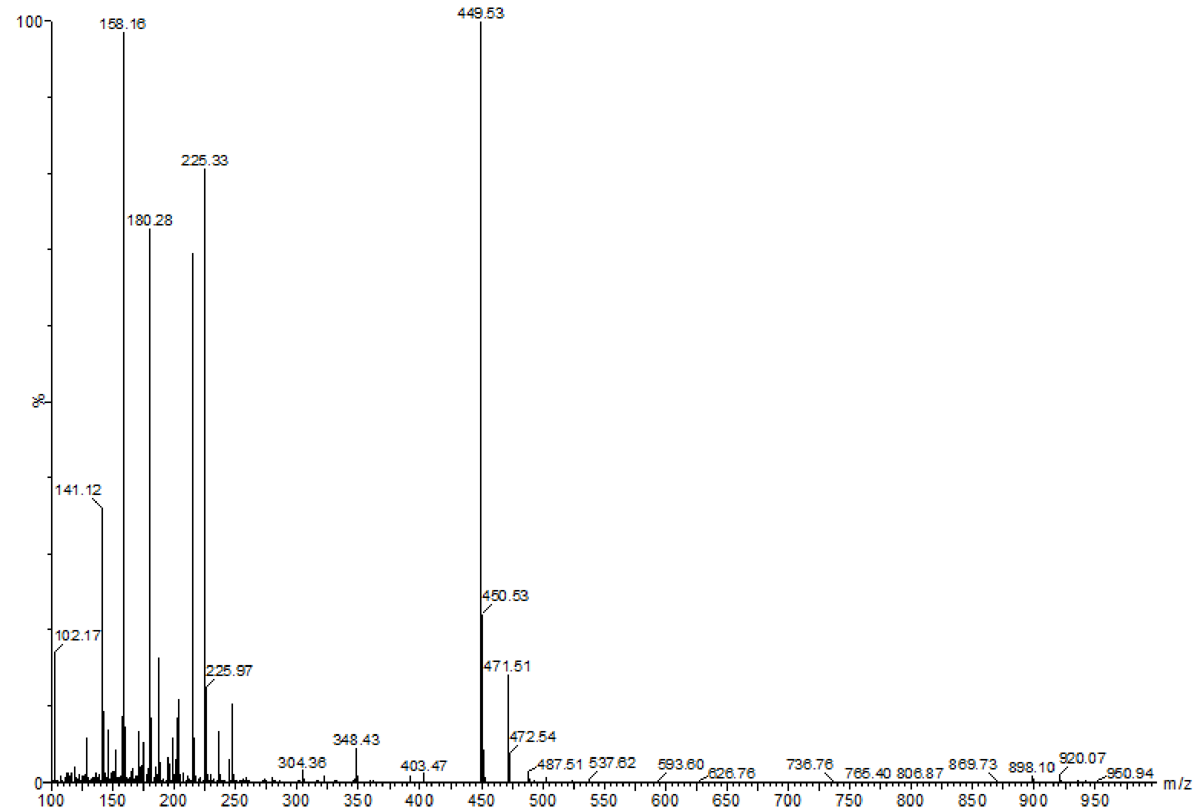

Fig. S13b ESI-MS spectrum of **L1**

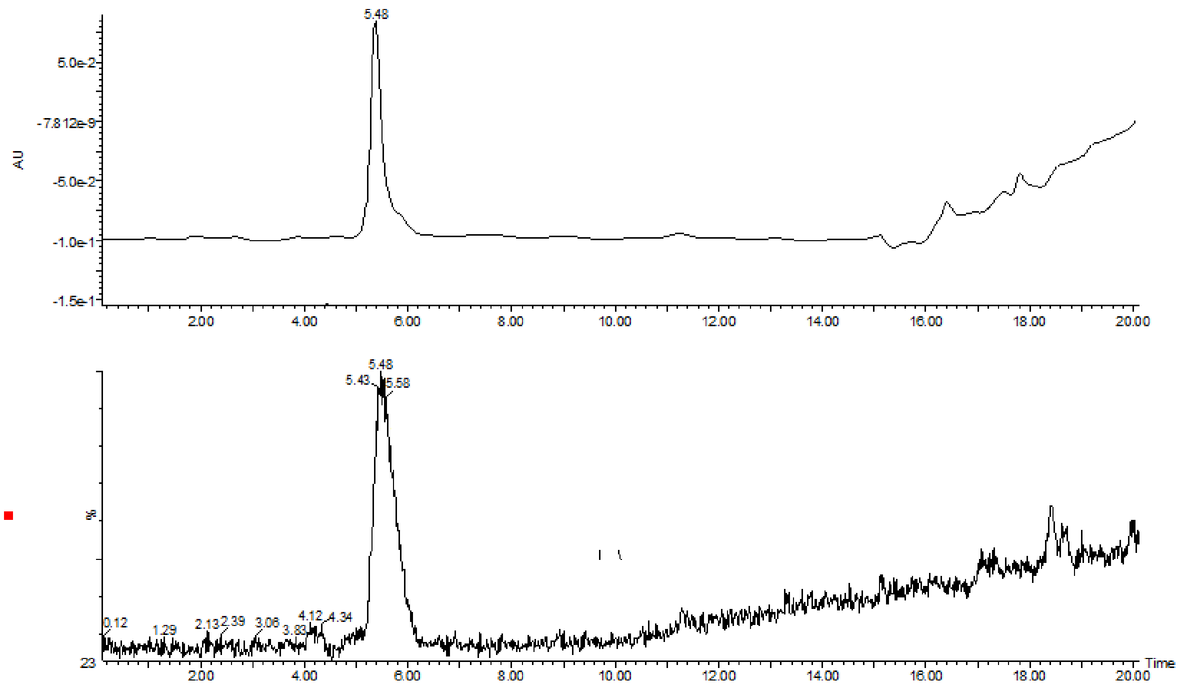

Fig. S14a HPLC-MS chromatogram of **L2**

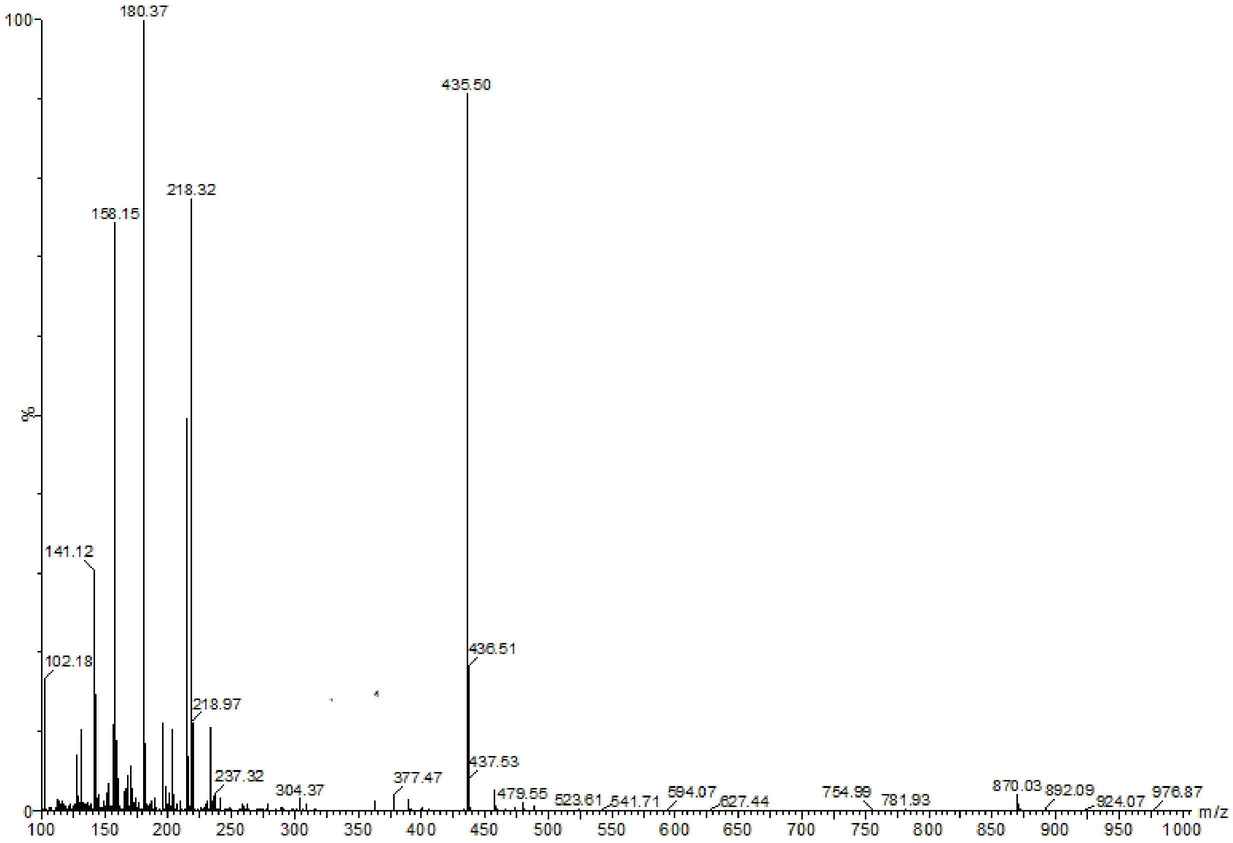

Fig. S14b ESI-MS spectrum of **L2**

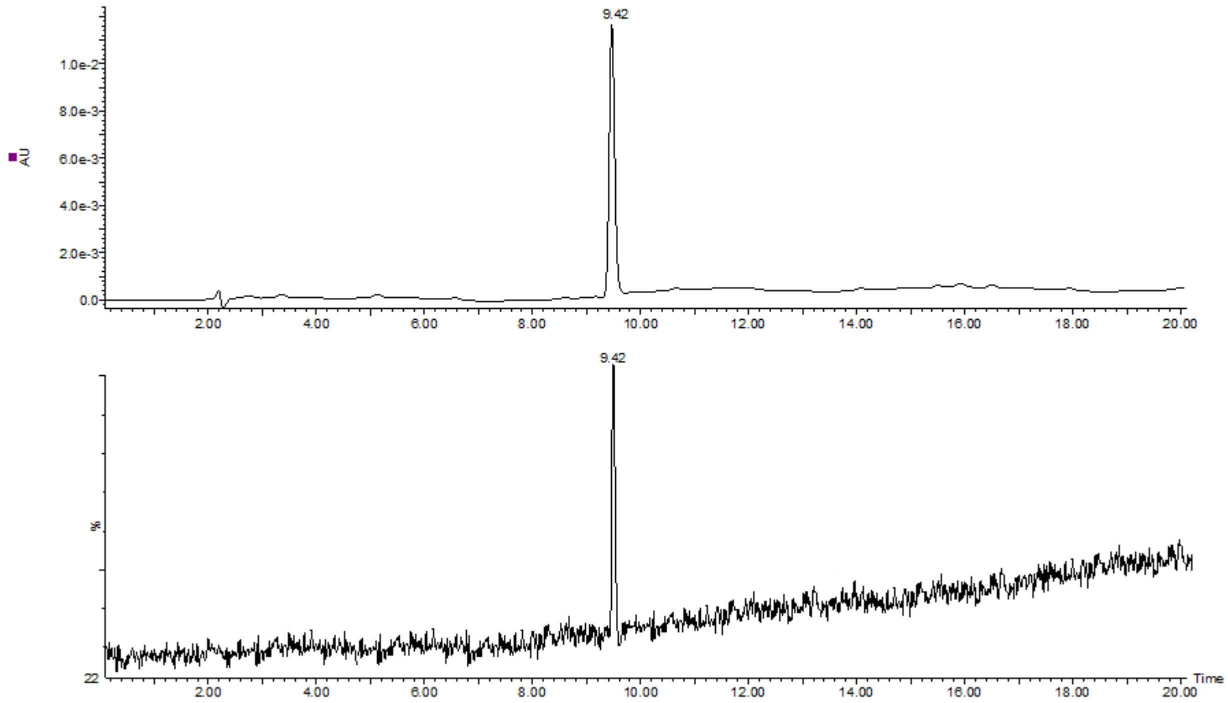

Fig. S15a HPLC-MS chromatogram of **GdL1**.

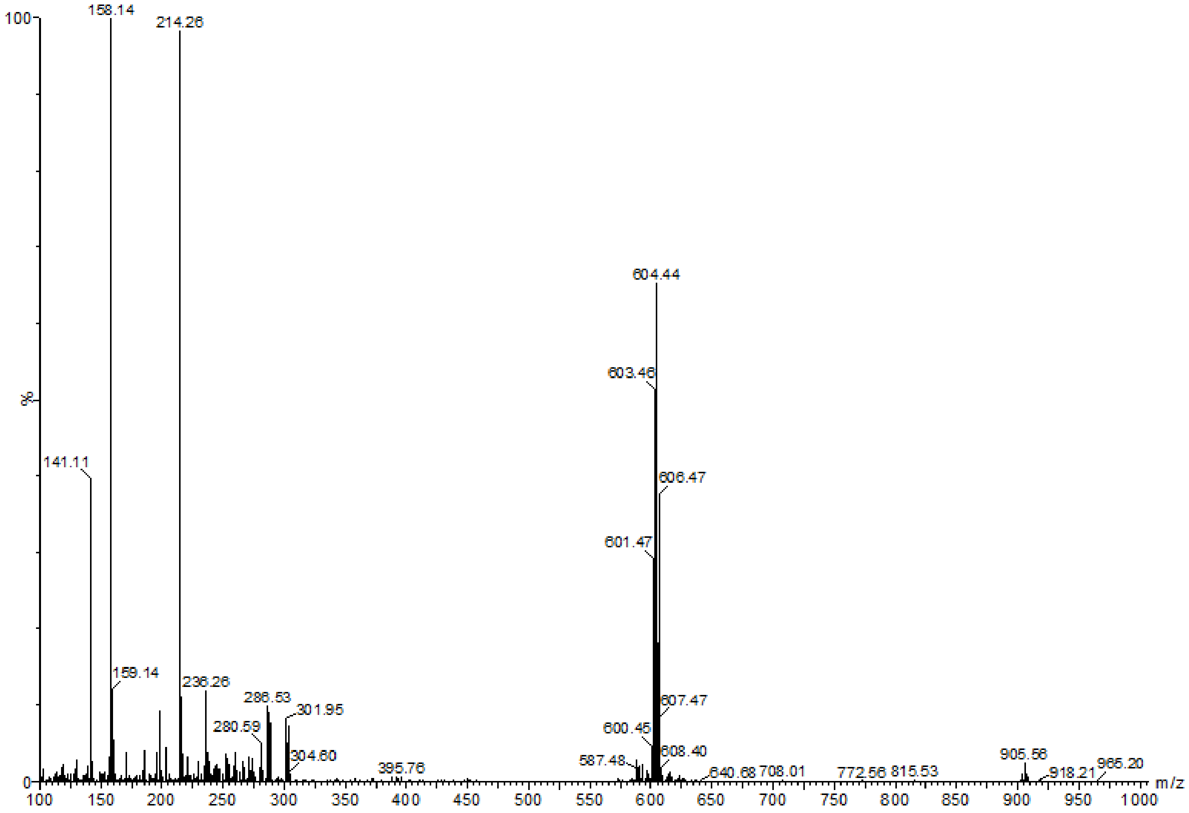

Fig. S15b ESI-MS spectrum of **GdL1**

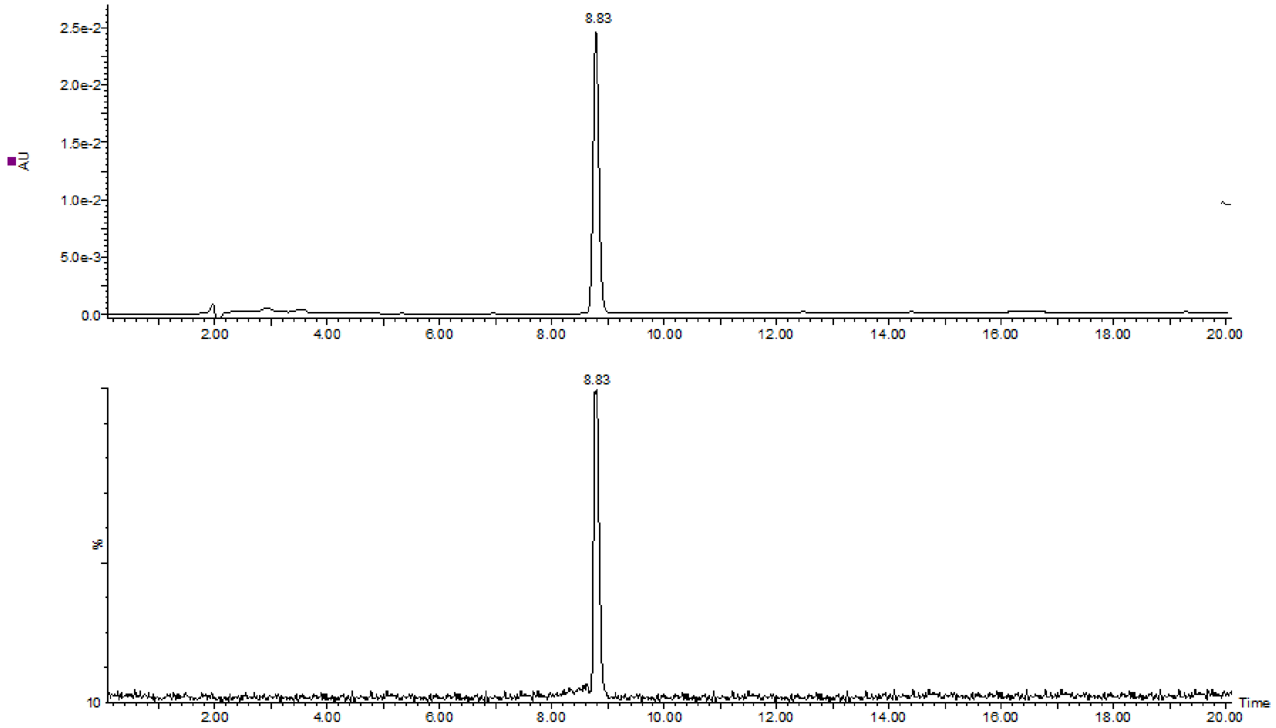

Fig. S16a HPLC-MS chromatogram of **GdL2**

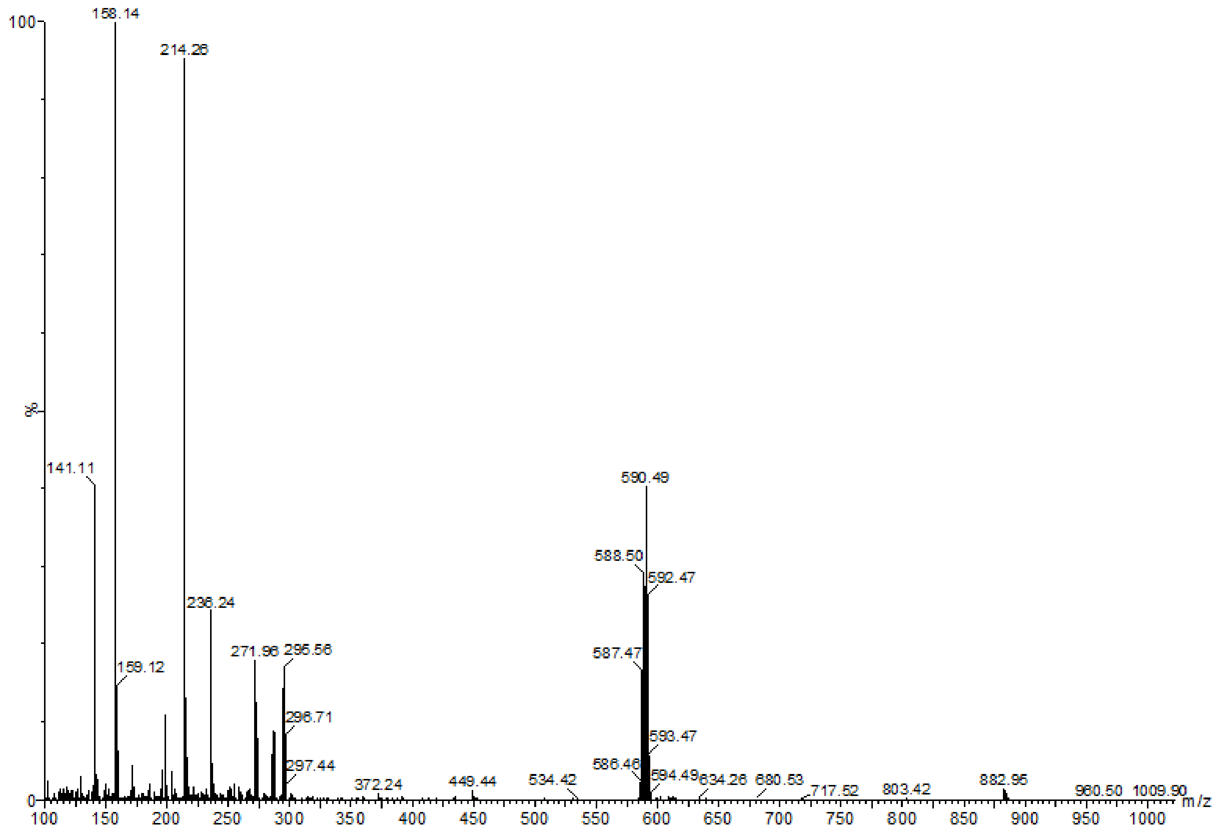

Fig. S16b ESI-MS spectrum of **GdL2**

5. References

1) Desreux, J. F.; Merciny, E.; Loncin, M. F., Nuclear Magnetic-Resonance and Potentiometric Studies of the Protonation Scheme of 2 Tetraaza Tetraacetic Macrocycles. *Inorg. Chem.* 1981, 20 (4), 987-991.

2) Bianchi, A.; Calabi, L.; Giorgi, C.; Losi, P.; Mariani, P.; Paoli, P.; Rossi, P.; Valtancoli, B.; Virtuani, M., Thermodynamic and structural properties of Gd3+ complexes with functionalized macrocyclic ligands based upon 1,4,7,10-tetraazacyclododecane. *J. Chem. Soc., Dalton Trans.* 2000, (5), 697-705.

3) Takacs, A.; Napolitano, R.; Purgel, M.; Benyei, A. C.; Zekany, L.; Brucher, E.; Toth, I.; Baranyai, Z.; Aime, S., Solution structures, stabilities, kinetics, and dynamics of DO3A and DO3A-sulphonamide complexes. *Inorg. Chem.* 2014, 53 (6), 2858-72.

4) Delgado, R.; da Silva, J. J., Metal complexes of cyclic tetra-azatetra-acetic acids. *Talanta* 1982, 29 (10), 815-22.

5) Tóth, É.; Király, R.; Platzek, J.; Radüchel, B.; Brücher, E., Equilibrium and kinetic studies on complexes of 10-[2,3-dihydroxy-(1-hydroxymethyl)-propyl]-1,4,7,10-tetraazacyclododecane-1,4,7-triacetate. *Inorg. Chim. Acta* 1996, 249 (2), 191-199.

6) Tóth, É.; Brücher, E.; Lázár, I.; Tóth, I., Kinetics of Formation and Dissociation of Lanthanide(Iii)-Dota Complexes. *Inorg. Chem.* 1994, 33 (18), 4070-4076.

7) Wu, S. L.; Horrocks, W. D., Kinetics of Complex-Formation by Macrocyclic Polyaza Polycarboxylate Ligands - Detection and Characterization of an Intermediate in the Eu3+-Dota System by Laser-Excited Luminescence. *Inorg. Chem.* 1995, 34 (14), 3724-3732.

8) Burai, L.; Fábián, I.; Király, R.; Szilágyi, E.; Brücher, E., Equilibrium and kinetic studies on the formation of the lanthanide(III) complexes, [Ce(dota)]- and [Yb(dota)]- (H4dota = 1,4,7,10-tetraazacyclododecane-1,4,7,10-tetraacetic acid). *J. Chem. Soc., Dalton Trans.* 1998, (2), 243-248.

9) Moreau, J.; Guillon, E.; Pierrard, J. C.; Rimbault, J.; Port, M.; Aplincourt, M., Complexing mechanism of the lanthanide cations Eu3+, Gd3+, and Tb3+ with 1,4,7,10-tetrakis(carboxymethyl)-1,4,7,10-tetraazacyclododecane (dota) - Characterization of three successive complexing phases: Study of the thermodynamic and structural properties of the complexes by potentiometry, luminescence spectroscopy, and EXAFS. *Chem. – Eur. J.* 2004, 10 (20), 5218-5232.

10) Perez-Malo, M.; Szabo, G.; Eppard, E.; Vagner, A.; Brucher, E.; Toth, I.; Maiocchi, A.; Suh, E. H.; Kovacs, Z.; Baranyai, Z.; Rosch, F., Improved Efficacy of Synthesizing *M(III)-Labeled DOTA

Complexes in Binary Mixtures of Water and Organic Solvents. A Combined Radio- and Physicochemical Study. *Inorg. Chem.* 2018, 57 (10), 6107-6117.
11) Leone, L.; Boccalon, M.; Ferrauto, G.; Fábián, I.; Baranyai Z.; Tei, L. Acid-catalyzed proton exchange as a novel approach for relaxivity enhancement in GdHPDO3A-like complexes, *Chem. Sci.*, 2020, 11, 7829–7835
12) Lattuada, L.; Horváth, D.; Colombo Serra, S.; Fringuello Mingo, A.; Minazzi, P.; Bényei, A.; Forgács, A.; Fedeli, F.; Gianolio, E.; Aime, S.; Giovenzana, G. B.; Baranyai Z. Enhanced relaxivity of GdIII-complexes with HP-DO3A-like ligands upon the activation of the intramolecular catalysis of the prototropic exchange. *Inorg. Chem. Front.*, 2021, 8, 1500-1510.
13) Kumar, K.; Tweedle, M. F.; Malley, M. F.; Gougoutas, J. Z., Synthesis, stability, and crystal structure studies of some Ca2+, Cu2+ and Zn2+ complexes of macrocyclic polyamino carboxylates. *Inorg. Chem.* 1995, 34 (26), 6472-6480.
14) Kumar, K.; Chang, C. A.; Francesconi, L. C.; Dischino, D. D.; Malley, M. F.; Gougoutas, J. Z.; Tweedle, M. F., Synthesis, Stability, and Structure of Gadolinium(III) and Yttrium(III) Macrocyclic Poly(amino carboxylates). *Inorg Chem* 1994, 33 (16), 3567-3575.
15) Clarke, E. T.; Martell, A. E., Stabilities of the alkaline earth and divalent transition metal complexes of the tetraazamacrocyclic tetraacetic acid ligands. *Inorg. Chim. Acta* 1991, 190 (1), 27-36.
16) Cacheris, W. P.; Nickle, S. K.; Sherry, A. D., Thermodynamic Study of Lanthanide Complexes of 1,4,7-Triazacyclononane-N,N',N"-Triacetic Acid and 1,4,7,10-Tetraazacyclododecane-N,N',N",N"'-Tetraacetic Acid. *Inorg. Chem.* 1987, 26 (6), 958-960.
